# Ecological structure and function in a restored versus natural salt marsh

**DOI:** 10.1371/journal.pone.0189871

**Published:** 2017-12-19

**Authors:** Ryan J. Rezek, Benoit Lebreton, Blair Sterba-Boatwright, Jennifer Beseres Pollack

**Affiliations:** 1 Department of Life Sciences, Texas A&M University - Corpus Christi, Corpus Christi, Texas, United States of America; 2 Littoral Environnement et Sociétés, CNRS - Université de La Rochelle, La Rochelle, France; 3 Department of Mathematics and Statistics, Texas A&M University - Corpus Christi, Corpus Christi, Texas, United States of America; Estacion Experimental de Zonas Aridas, SPAIN

## Abstract

Habitat reconstruction is commonly employed to restore degraded estuarine habitats and lost ecological functions. In this study, we use a combination of stable isotope analyses and macrofauna community analysis to compare the ecological structure and function between a recently constructed *Spartina alterniflora* salt marsh and a natural reference habitat over a 2-year period. The restored marsh was successful in providing habitat for economically and ecologically important macrofauna taxa; supporting similar or greater density, biomass, and species richness to the natural reference during all but one sampling period. Stable isotope analyses revealed that communities from the natural and the restored marshes relied on a similar diversity of food resources and that decapods had similar trophic levels. However, some generalist consumers (*Palaemonetes* spp. and *Penaeus aztecus*) were more ^13^C-enriched in the natural marsh, indicating a greater use of macrophyte derived organic matter relative to restored marsh counterparts. This difference was attributed to the higher quantities of macrophyte detritus and organic carbon in natural marsh sediments. Reduced marsh flooding frequency was associated with a reduction in macrofaunal biomass and decapod trophic levels. The restored marsh edge occurred at lower elevations than natural marsh edge, apparently due to reduced fetch and wind-wave exposure provided by the protective berm structures. The lower elevation of the restored marsh edge mitigated negative impacts in sampling periods with low tidal elevations that affected the natural marsh. The results of this study highlight the importance of considering sediment characteristics and elevation in salt marsh constructions.

## Introduction

Coastal salt marshes are among the most important habitats on earth in terms of ecosystem service provision [1]. Salt marsh (hereafter “marsh”) habitats are highly productive and provide fishery support, water purification, coastal protection, and carbon sequestration [2]. The complex structure formed by marsh plants provides essential refuge habitat and feeding grounds for juvenile fish and crustacean species [3]. In the Gulf of Mexico (GOM), marshes support economically important fisheries through the provision of refuge and trophic support for taxa such as penaeid shrimp and blue crab [4].

Marshes in the GOM have experienced severe habitat loss and degradation relative to historic levels. Major drivers of marsh degradation include coastal development, agricultural land use, dredging, hydrologic alterations, and other anthropogenic impacts [5]. As a result, approximately 50% of coastal marsh habitat in GOM states has been lost between 1780 and 1980 [6].

Marsh restoration is an important tool for enhancing ecological functions and mitigating habitat loss in degraded coastal systems. Marsh restoration techniques include the restoration of previously restricted tidal regimes [7], invasive species removal [8], and the use of dredged or excavated material to construct marshes [9]. Marsh construction has become a particularly common restoration method in urban coastal regions, and is often used to offset habitat losses associated with coastal development [10].

The general goal of habitat restoration is to recover the ecological structure and function of natural habitats they are intended to recreate [11]. Post-restoration monitoring is essential for evaluating the ecological success of restoration projects and improving restoration practices [12]. Post-restoration monitoring frequently includes assessing metrics of ecological structure (e.g. species composition, abundance) in restored habitats in comparison to a natural reference. However, the recovery of ecological functions, such as nutrient cycling and trophic pathways supporting secondary production, are rarely evaluated [13,14].

Stable isotope based food web analysis is becoming an increasingly common technique used in studying aspects of functional recovery and equivalence in restored coastal habitats [8,15,16]. Primary producer carbon isotopic composition varies in relation to environmental and physiological factors associated with photosynthesis. The isotopic composition of carbon changes little as it moves through the food web and can be used to trace organic matter in consumer tissue to its primary producer source [17]. The isotopic composition of nitrogen undergoes a predicable step-wise enrichment in ^15^N with trophic transfers (2–4‰) and can be used to determine consumer trophic levels [17,18]. These properties permit the study of important ecological functions related to trophic structure. In the context of restoration monitoring, stable isotope analysis can be used to examine the recovery of important trophic linkages driving secondary production and trophic diversity.

In this study, we examine the structural and functional characteristics of a recently constructed marsh in comparison to a natural reference marsh to evaluate the short-term ecological success of the restoration (4 to 5 years post-restoration). We employ a system-wide approach of evaluating traditional metrics of community structure in combination with stable isotope based food web analysis in each habitat taking into account tidal inundation and sediment organic matter characteristics, as these parameters have been shown to influence aspects of marsh community structure and functions [9,19–21].

## Materials and methods

### Study site

**Fig 1:**
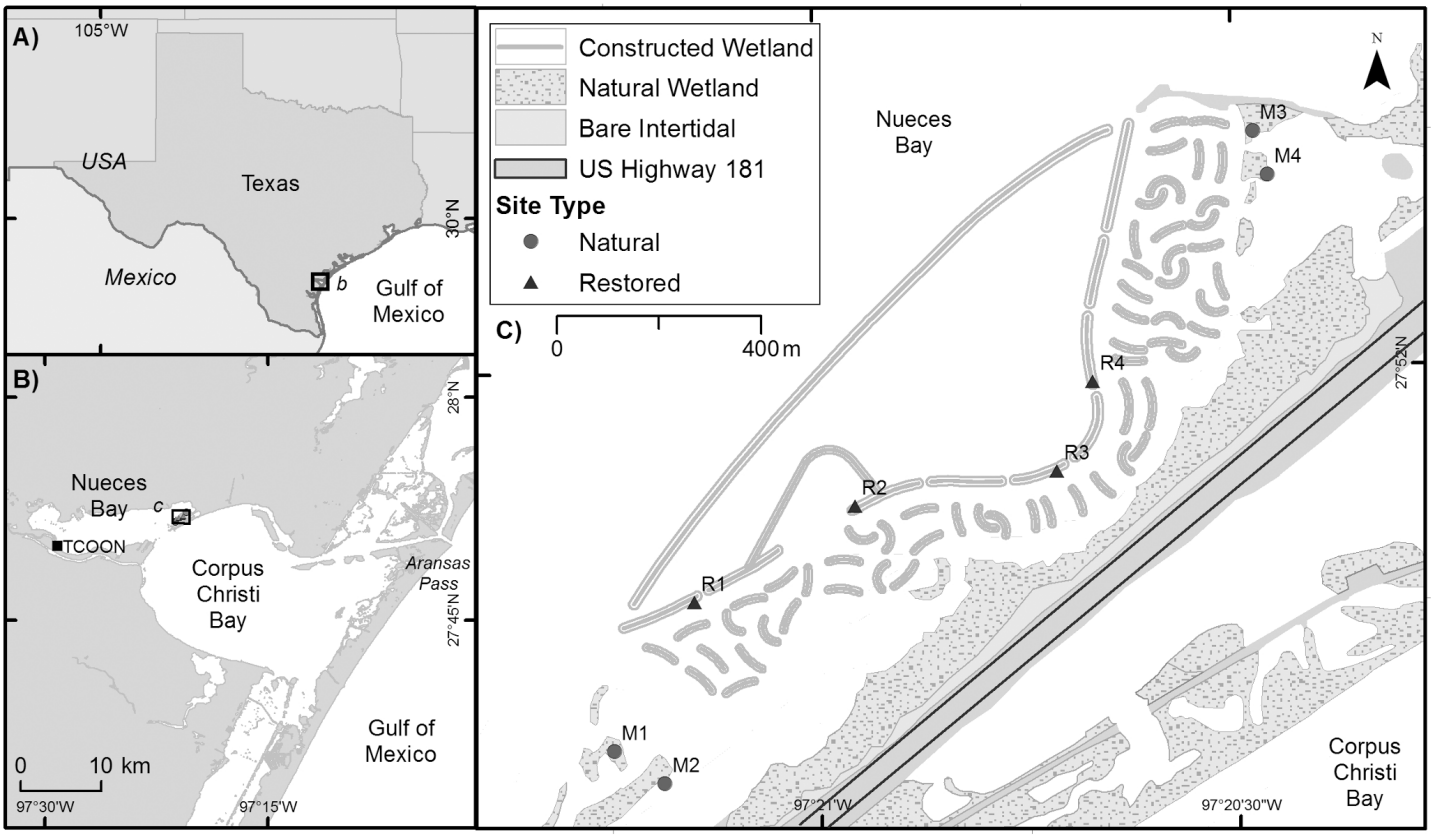
Restored and natural reference marsh sites. Map of study region (A) and study site with labeled restored and natural salt marsh sampling sites (B) in Nueces Bay, Texas. TCOON: Texas coastal ocean observation network.

Nueces Bay is a shallow subtropical estuary located in the Texas Coastal Bend, U.S.A. This secondary bay drains into Corpus Christi Bay which is connected to the Gulf of Mexico through Aransas Pass (Fig 1A). The surface area of Nueces Bay is 7,475 ha with an average depth of 0.7 m at mean low tide. Mean tide level in Nueces Bay is 0.242 m above NAVD 88 with an average tidal range of 0.12 m [22]. The bay receives most of its freshwater from the Nueces River and relatively little inflow outside of storm-related discharge events [23]. Nueces Bay supports approximately 24 ha of low marsh *Spartina alterniflora* (hereafter “*Spartina*”) habitat which provides food and nursery habitat for economically important species [24,25]. Mid marsh vegetation in the study area was composed primarily of *Batis maritima* and *Salicornia bigelovii*.

Approximately 100 ha of marsh habitat were lost along the eastern margin of Nueces Bay during the construction of the Portland Causeway on U.S. Highway 181 in the late 1940’s and through subsequent erosion [15]. To compensate for this habitat loss, the Coastal Bend Bays and Estuaries Program (one of 28 areas in the National Estuary Program) initiated a marsh restoration project in 2011. The project created ~29 ha of salt marsh complex habitat consisting of protective berms and terraces planted with *Spartina* (25%) as well as protected open water (75%) (Fig 1B). Berms and terraces were constructed by mechanically side casting sediment from borrow trenches to create a surface ~ 3 m wide at 0.8 to 1.2 m elevation (NAVD 88) with ~ 6 m buffer zone from trenches.

### Field sampling and measurements

Macrofauna (fish and decapod crustaceans) were sampled in *Spartina*-dominated marsh edge habitat at four sites within restored and natural habitats in Nueces Bay (Fig 1B). Sampling was conducted seasonally during spring (May 27^th^) and summer (Aug 13^th^) of 2014 and winter (Feb 16^th^), spring (May 18^th^), and summer (Aug 17^th^) of 2015. Macrofauna were sampled at each site with triplicate tows of a modified epibenthic sled equipped with a 1-mm mesh conical plankton net (0.6 m width x 0.8 m height). Sampling sites were selected to occur on separate “islands” surrounded by open water. All 4 natural marsh islands in the area were sampled (i.e. one sampling site per island). Among the 8 restored marsh berm islands, 4 were randomly selected as sampling sites. All sites supported sufficient *Spartina* coverage at the beginning of the study for sampling. The sled was pulled by hand along the fringe of *Spartina* marsh edge for 8.4 m, sampling an area of 5 m^2^ (0.6 m width • 8.4 m tow). *Spartina* shoot counts were conducted at each site using triplicate 0.25 m^2^ quadrats, samples were taken in the marsh edge (seaward 1 m of *Spartina*) within the length of each sled tow. Cores (35.4 cm^2^) were used to collect above and below ground (0–20 cm) biomass of a randomly selected *Spartina* culm within each quadrat. A total of 120 *Spartina* (biomass/shoot count) and macrofauna community samples (3 replicates • 4 sites • 5 sampling periods • 2 marsh types) were taken over the course of the study. All necessary collection permits were obtained from Texas Parks and Wildlife Department (Permit SPR-0911-344). No endangered species were collected in this study. Following approved Institutional Animal Care and Use Committee of Texas A&M University-Corpus Christi guidelines (IACUC #08–14), euthanasia occurred via rapid chilling (hypothermic shock) in an ice slurry in a cooler.

Suspended particulate organic matter (SPOM) was sampled with two replicate bottom water collections 0.1 m above the sediment-water interface at 3 sites (M2, M4 and R3; Fig 1). Water was sieved through a 250-μm screen to remove large zooplankton and particles and then filtered through pre-combusted (450°C, 4 hours) Whatman GF/F filters (0.7-μm nominal pore size). Surface sediment (0–2 cm) was collected using cores (35.4 cm^2^) on the marsh edge fringe at all sites. Nueces Bay tidal elevation data (in 30-minute intervals) from a nearby long-term data collection station were provided by the Conrad Blucher Institute for Surveying and Science at Texas A&M University Corpus Christi as part of the Texas Coastal Ocean Observation Network (TCOON) (http://www.cbi.tamucc.edu/TCOON). Three elevation measurements were taken along the marsh edge at the seaward *Spartina* fringe in each site in locations sampled with the epibenthic sled, using a real-time kinematic global positioning system (Altus Positioning Systems, Torrance, California). Marsh edge elevation data were related to water level data to create an index of flooding duration to estimate the proportion of time the marsh edge was flooded with at least 5 cm of water during the month prior to each sampling period using the equation: flood duration index (%) = ((# 0.5 hrs. water level > 5 cm + marsh edge elevation) month^-1^ / # 0.5 hrs. month^-1^) • 100%. Five centimeters was considered the threshold in which the marsh edge was functionally accessible to macrofauna species.

### Stable isotope analysis

All samples were transported to the laboratory in ice chests with ice packs. Subsamples of each macrofauna species, 3 to 6 individuals if available, were selected for stable isotope analyses. All other macrofauna were fixed in buffered 10% formalin for abundance, richness, and biomass assessments. Organisms were enumerated and identified to the lowest practical taxonomic level. Live *Spartina* above- and below-ground materials collected in cores were rinsed thoroughly with tap water to remove detrital matter. Sediment samples were sieved through a 500-μm screen to separate macrodetritus from surface sediment samples. *Spartina* above- and below-ground material, sediment macrodetritus and macrofauna were dried for 24 hours at 55°C to obtain dry weight biomass.

For stable isotope analyses, small macrofauna (< 10 mm) were kept alive for 24 hours in artificial seawater to empty gut contents and whole individuals were used for analysis. Larger macrofauna were dissected to obtain muscle tissue. All flora and fauna samples were rinsed with deionized water and then stored at -20°C prior to analysis. Epiphytic microalgae were removed from *Spartina* stems with a scalpel and sorted under a dissecting microscope to remove detritus, macroalgae and macro/meiofauna. Stable isotope analysis was conducted on sieved (< 500 μm) sediment samples to determine the isotopic composition of surface sediment organic matter (SSOM) and on remaining sediment macrodetritus (> 500-μm). All samples were freeze-dried. Tissue samples were ground into a homogeneous powder using a ball mill and SSOM samples were ground using a mortar and pestle. Samples possibly containing inorganic carbonates (e.g. SPOM, SSOM, algal epiphytes and small crustaceans) were acidified prior to stable isotope analysis. Filters containing SPOM were decarbonated by contact with HCl fumes under light vacuum for 4 hours. All other samples were decarbonated with 1 mol l^-1^ HCl added drop by drop until cessation of bubbling. To avoid bias in δ^15^N measurements due to acidification, δ^15^N and δ^13^C measurements were carried out on raw and decarbonated samples, respectively. Sediment organic carbon (OC) content was determined from acidified SSOM samples.

Samples were precisely weighed (± 1 μg), encapsulated in combustion cups and carbon/nitrogen isotopic compositions were determined using a Costech ECS4010 elemental analyzer (Valencia, CA) connected to a continuous flow Thermo Delta V Plus isotope ratio mass spectrometer via a Thermo Conflo IV interface (Bremen, Germany). Replicate analyses of isotopic standard reference materials USGS 40 (δ^13^C = -26.39 ‰; δ^15^N = -4.52 ‰) and USGS 41 (δ^13^C = 37.63 ‰; δ^15^N = 47.57 ‰) were used to normalize preliminary isotopic values to the air (δ^15^N) and Vienna Pee Dee Belemnite (δ^13^C) scales. Isotope values are expressed in δ notation following the formula δX (‰) = [(R_sample_ */* R_standard_)– 1] • 10^3^, where X is ^13^C or ^15^N and R is ^13^C/^12^C or ^15^N/^14^N isotopic ratio, respectively. Methionine standards (Costech) were analyzed after every 12 samples to monitor instrument performance and check data normalization. The precision of the laboratory standards was ± 0.2‰ for carbon and nitrogen.

### Statistical analysis

All statistical analysis was conducted in the R statistical software environment [26]. Differences in *Spartina* above- and below-ground biomass, density, and macrofauna species richness, density and biomass between the restored and natural marsh within sampling periods and between sampling periods within each marsh were analyzed with linear mixed effects two-way analysis of variance (ANOVA) models using the nlme package in R [27]. Marsh type and season were used as fixed effects with site as a random effect with a random intercept to account for spatial dependence within sites and across sampling periods [28]. For each parameter, a two-way ANOVA was fit using methods outlined in Zuur [28]. Planned contrasts were established *a priori* and applied to these models to compare parameters between marsh types within each sampling period (fixed effects ANOVA degrees of freedom 1, 22) and to compare parameters within each marsh between sampling periods (fixed effects ANOVA degrees of freedom 4, 55). This hypothesis framework was used to detect potential changes in between-marsh parameter relationships over time due to successional processes in the restored marsh. Since the contrasts focus on the specific hypotheses of interest, omnibus F-test results are not reported. *P*-values for within-marsh sampling period contrasts were adjusted for multiple comparisons using Westfall’s modification of Tukey’s HSD test with the multcomp R package [29]. When significant differences were found, the *P*-value reported for contrasts represents the maximum *P*-value below 0.05. Assumptions of normality were assessed with Shapiro–Wilk tests and homoscedasticity was inspected with normalized residual *vs*. fitted value plots. *Spartina* below-ground biomass data were square root transformed to meet assumptions of residual normality. Models were compared and selected based on corrected Akaike information criterion (AICc). Due to over-dispersion, *Spartina* and macrofauna density data were analyzed using generalized linear mixed effects two-way ANOVA models (negative binomial distribution) with the lme4 R package [30].

A Bray-Curtis similarity matrix of log(*y*+1) transformed of macrofauna species density data was used for multivariate community analysis and presented with a non-metric multidimensional scaling (nMDS) plot using the vegan R package [31]. Multivariate macrofauna similarity between marsh types within each sampling period was evaluated with permutational multivariate analysis of variance tests (PERMANOVA, 9,999 permutations) [32]. Community compositional heterogeneity was compared between marsh types using distance-based tests of homogeneity of multivariate dispersions [33] using the betadisper function. Four empty epibenthic sled samples, 2 within each marsh type during winter 2015, were omitted from multivariate analysis.

Spatial variation in isotopic compositions (i.e. natural *vs*. restored) of potential food sources was analyzed using Wilcoxon rank sum tests and temporal variation was assessed using Kruskal-Wallis rank sum test with post-hoc multiple comparisons performed with Dunn’s test [34] using Holm’s p-value adjustment procedures with the dunn.test R package [35]. Decapod δ^13^C values and trophic levels (TLs) were compared between marshes with Wilcoxon rank sum tests. As marsh edge dwelling macrofauna generally exhibit high site fidelity outside of ontogenetic migrations [36–38], consumer isotopic compositions were expected to reflect the assimilation of locally available resources within each marsh type. Isotopic compositions of benthic diatoms in the Nueces Estuary were characterized by [25] and included as a potential food source in this study.

Consumer TLs (TL_i_) were calculated based on the difference between δ^15^N values of consumer and the average of major primary producer baselines using

$$\text{T}{\text{L}}_{i}=1+\frac{\left(\delta^{15}{\text{N}}_{i}-\delta^{15}{\text{N}}_{b}\right)}{\text{TFF}}$$ (1)

where δ^15^N_*i*_ is the δ^15^N value of consumer *i* and δ^15^N_*b*_ is the δ^15^N value of the baseline. A trophic fractionation factor (TFF) of 3.4‰ from literature was applied [17,18]. Averaged δ^15^N values of SPOM, *Spartina* (leaves/stems and roots), *Spartina* epiphytic microalgae, and benthic diatoms were used as baselines to calculate TLs. δ^13^C, δ^15^N and TL means are given with ± standard deviation, all other means are given with ± standard error.

Stable isotope mixing models (SIMMs) were run to estimate the contribution of selected primary resources as food sources of dominant decapod consumers (*Palaemonetes spp*., *Penaeus aztecus* and *Callinectes sapidus*) within each marsh during each sampling period using the simmr R package [39]. SIMMs were used to estimate the contribution of SPOM, *Spartina* epiphytic microalgae, *Spartina* (stems/leaves and roots) and benthic diatoms. TFFs used for SIMMs were 3.4 ± 0.4‰ for δ^15^N and 0.3 ± 1.3‰ for δ^13^C [18,40]. The models were run for 10^4^ iterations and the first 1,000 iterations were discarded. The source posterior distribution median was used as an unbiased estimate of dietary proportion; 95% and 50% credible intervals are also reported.

The effect of flood duration on average site spring and summer macrofauna biomass was analyzed with linear regression. Biomass data was log(y+1) transformed for this analysis to meet assumptions of residual normality. The relationship between flood duration and spring and summer TLs of dominant decapods (*Palaemonetes* spp., *P*. *aztecus* and *C*. *sapidus*) from both marshes were also evaluated with linear regression.

## Results

### Habitat characteristics

Mean water temperature ranged from 12.0 to 30.7°C during sampling periods, with little spring and summer inter-annual variation (≤ 1°C) (Table 1). Salinity was 12.5 and 15.7 psu lower in spring and summer of 2015, respectively, than in spring and summer of 2014. Over the course of the survey, quantities of sediment OC in the natural marsh were from 2.4 to 5.1 times greater than in the restored marsh. Quantities of sediment N in the natural marsh were from 2.5 to 6.0 times greater than in the restored marsh (Table 1). Surface sediment in the natural marsh (0–2 cm) contained from 17 to 273 times as much dry weight detritus (g m^-2^) than the restored marsh over the course of the study (Table 1). Surface sediment OC, N and macrodetritus were greater in the natural marsh during all periods sampled (Table 1).

**Table 1:** Sediment and hydrographic data.

	Marsh	Spring 2014	Summer 2014	Winter 2015	Spring 2015	Summer 2015
**Sediment organic C (%)**	Nat	0.81±0.27	0.45±0.13	-	0.46±0.15	0.52±0.11
	Rest	0.16±0.03	0.19±0.04	-	0.22±0.04	0.11±0.01
**Sediment N (%)**	Nat	0.10±0.03	0.05±0.01	-	0.06±0.01	0.06±0.01
	Rest	0.02±<0.01	0.02±0.01	-	0.03±0.01	0.01±<0.01
**Sediment macrodetritus**	Nat	218.5±57.5	50.6±18.8	-	49.4±16.9	53.1±17.8
	Rest	0.8±0.2	0.9±0.5	-	2.3±1.7	3.2±1.8
**Salinity (psu)**	Both	35.4±0.2	40.1±0.2	32.5±0.1	22.9±0.1	24.4±0.4
**Temp. (°C)**	Both	26.8±0.8	30.7±0.8	12.0±0.3	27.8±0.7	30.3±1.0

Sediment and hydrographic data (mean ± standard error) from natural (Nat) and restored (Rest) marsh sites in Nueces Bay, Texas. Sediment macrodetritus (> 500-μm) is represented in g dry weight m^-2^, no data indicated with *hyphen*. Mean salinity and water temperature (Temp.) values from samples at sites M2, R3 and M4.

**Fig 2:**
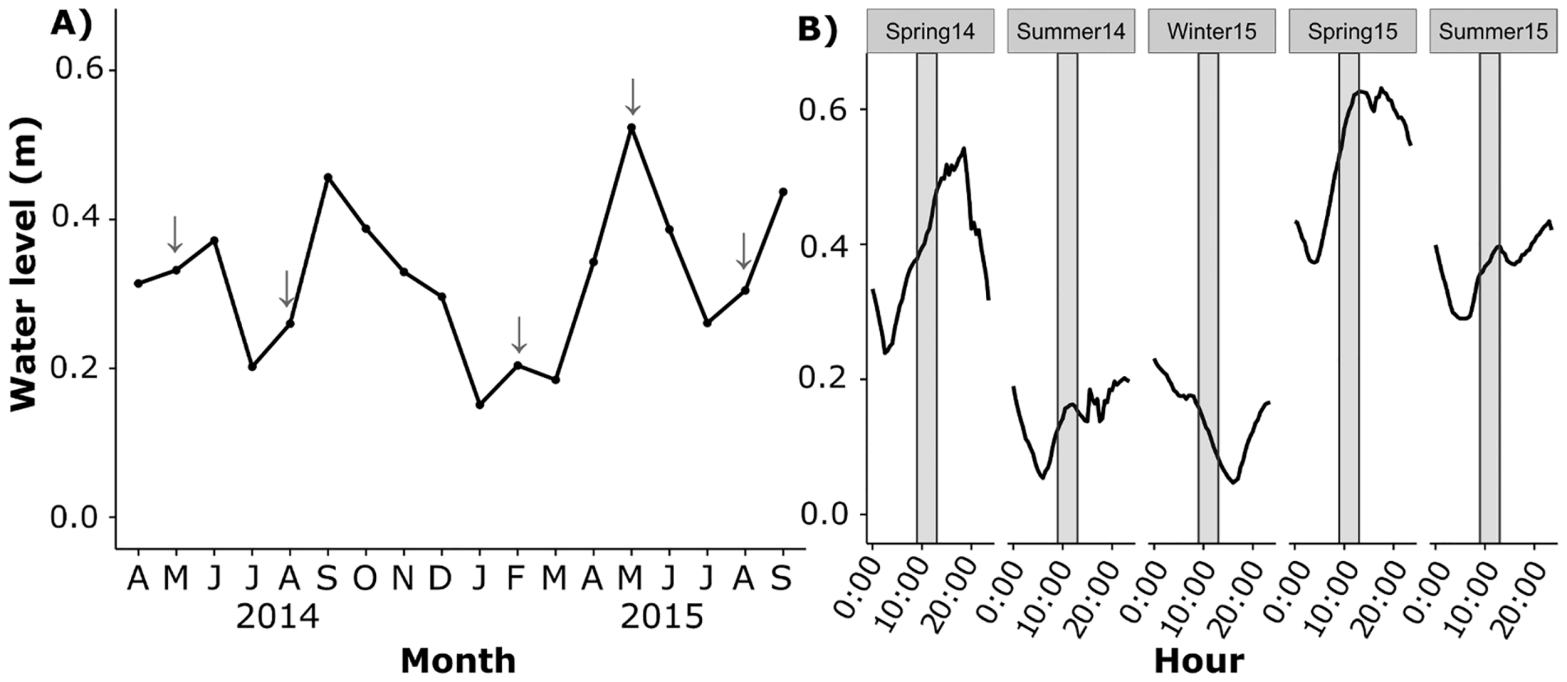
Water level during study and sampling periods. Mean monthly water level (NAVD 88) throughout the study duration (sampling months indicated with arrows) (A) and water level during sampling days (approximate sampling duration indicated with grey boxes) (B) in Nueces Bay, Texas.

**Fig 3:**
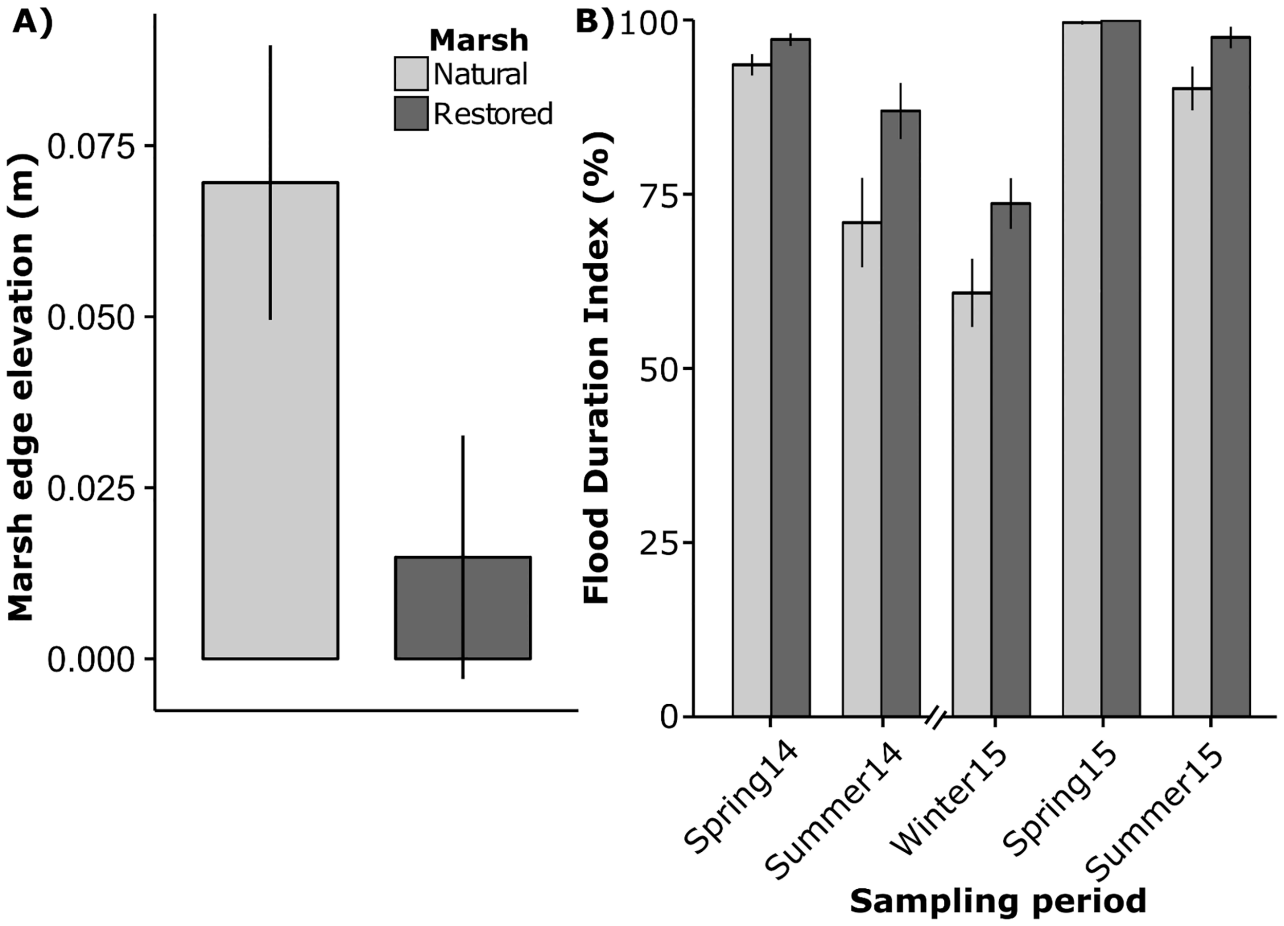
Marsh elevation and flood duration index. Mean ± standard error of marsh edge elevation (NAVD 88) in restored and natural marsh sites (A) and marsh edge flood duration (time water level > 5 cm + marsh elevation • total time^-1^) within one month prior to sampling period (B).

Mean monthly water level in Nueces Bay during the study period ranged from 0.15 m in January 2015 to 0.52 m (NAVD 88) in May 2015 (Fig 2A). Mean water level during spring and summer sampling months was greater during 2015 than in 2014, with May 2015 0.19 m higher and August 2015 0.05 m higher than 2014 averages. The average water level during marsh sampling was 0.15 m in summer 2014 and 0.12 m in winter 2015 (Fig 2B). The average water level during marsh sampling ranged from 0.38 to 0.59 m during all other sampling periods. The mean marsh edge elevation in natural mash sites averaged 0.070 ± 0.020 m compared to 0.015 ± 0.018 m (NAVD 88) in the restored marsh (Fig 3A); or -0.172 m and -0.227 m relative to mean tide elevation, respectively. Mean marsh edge flooding duration in the natural marsh sites ranged from 61% in winter 2015 to 100% in spring 2015, and 74% in winter 2015 to 100% in spring 2015 in the restored marsh (Fig 3B). The lowest flood durations outside of winter occurred in summer of 2014, with mean flooding duration of 71% in the natural marsh and 87% in the restored marsh.

**Fig 4:**
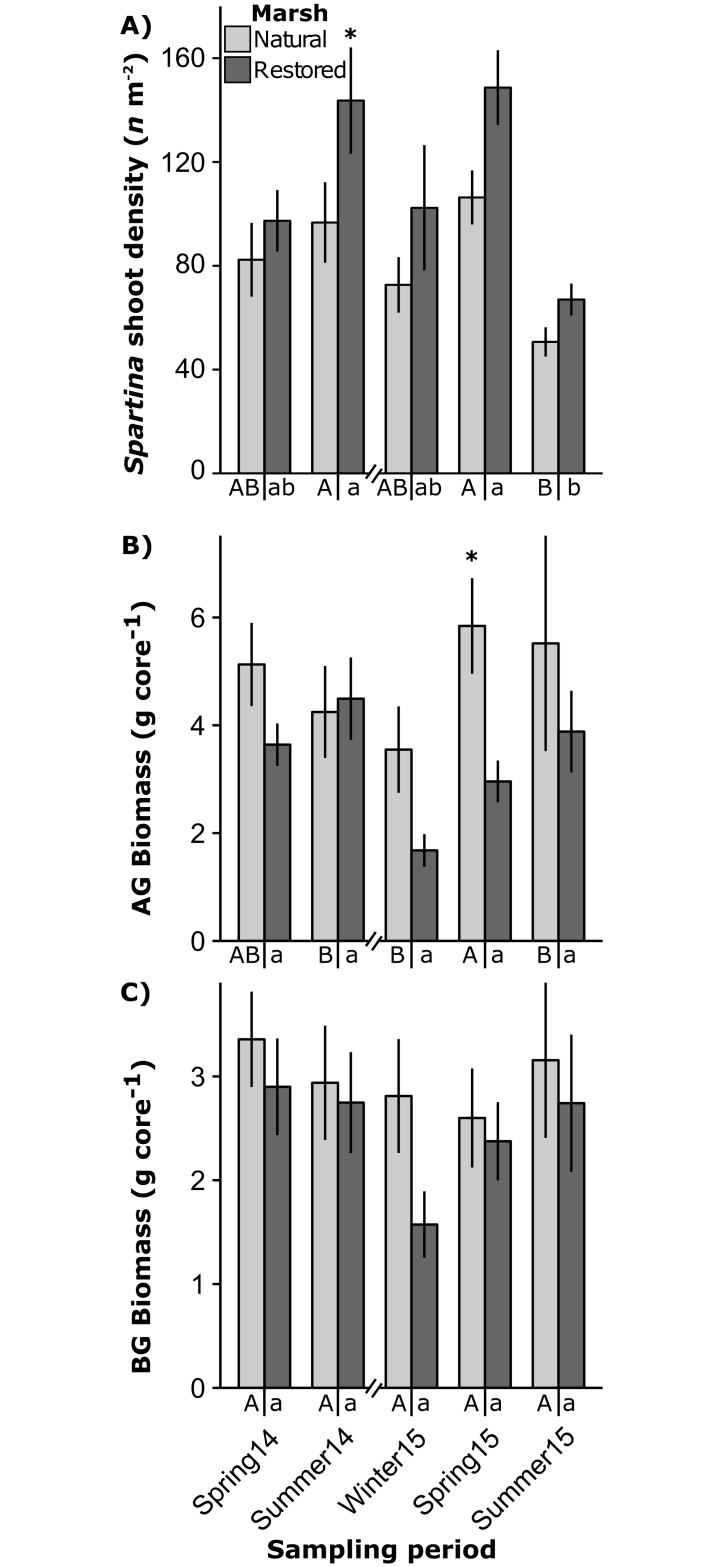
*Spartina* density and biomass. Mean ± standard error of *Spartina* shoot density (A), above ground (AG) biomass (B) and below ground (BG, 0–20 cm) biomass (dry weight) (C) of *Spartina* per 35.4 cm^2^ core in restored and natural marsh sites in each sampling period in Nueces Bay, Texas. Significant differences between restored and natural marshes within seasons indicated by *, and within marsh seasonal contrast groupings indicated under x-axis (ANOVA contrasts *P* < 0.05).

In the natural marsh edge, mean *Spartina* density varied seasonally (ANOVA contrasts, *P* ≤ 0.006) ranging from 50.7 ± 5.6 *n* m^-2^ in summer 2015 to 106.3 ± 10.4 *n* m^-2^ in spring 2015 (Fig 4A). Restored marsh edge *Spartina* density also varied seasonally (ANOVA contrasts, *P* < 0.001) ranging from 67.0 ± 6.2 *n* m^-2^ in summer 2015 to 148.7 ± 14.4 in *n* m^-2^ in spring 2015. *Spartina* density was similar between marsh types in all seasons except for summer 2014, where the restored marsh had higher densities of *Spartina* than the natural marsh (ANOVA contrast, *P* = 0.040) (Fig 4A). In the natural marsh, *Spartina* above-ground biomass ranged from 3.6 ± 0.8 g core^-1^ in winter 2015 to 5.8 ± 0.9 g core^-1^ in spring 2015 (35.4 cm^2^ core) and varied significantly between sampling periods (ANOVA contrasts, P ≤ 0.012) (Fig 4B). Natural marsh *Spartina* below-ground biomass (0–20 cm) ranged from 2.6 ± 0.5 g core^-1^ in spring 2015 to 3.4 ± 0.5 g core^-1^ in spring 2014 and was similar between sampling periods (Fig 4C). Restored marsh *Spartina* above- and below-ground biomass were stable between sampling periods; with above-ground biomass ranging from 1.6 ± 0.3 g core^-1^ in winter 2015 to 4.5 ± 0.8 g core^-1^ in summer 2014 and below-ground biomass ranging from 1.6 ± 0.3 g core^-1^ in winter 2015 to 2.9 ± 0.6 g core^-1^ in spring 2014 (Fig 4B and 4C). *Spartina* above-ground biomass was significantly greater in the natural marsh in spring 2015 (ANOVA contrast, *P* = 0.029) (Fig 4B).

### Macrofauna community

**Fig 5:**
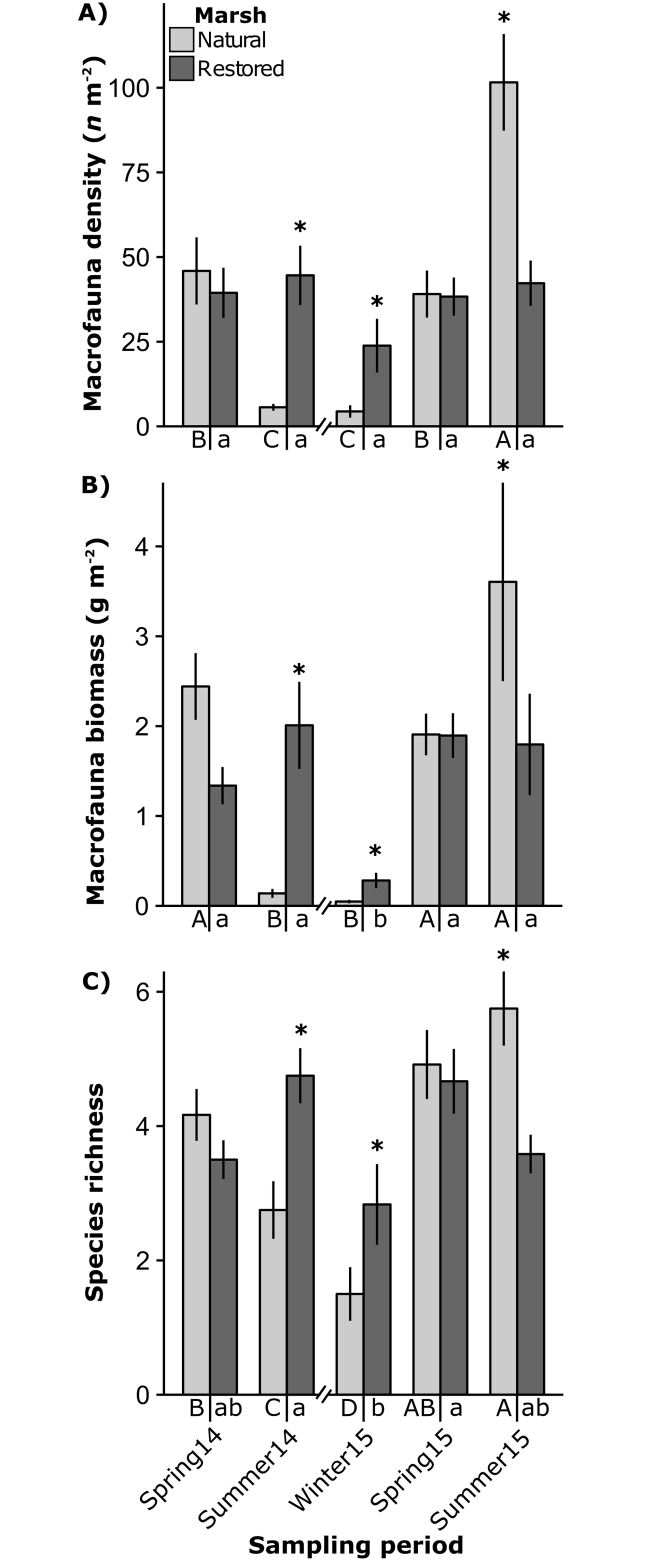
Macrofauna density, biomass, and richness. Mean ± standard error of macrofauna density (A), macrofauna biomass (dry weight) (B) and macrofauna species richness (C) in restored and natural marsh habitats during each sampling period in Nueces Bay, Texas. Significant differences between restored and natural marshes within seasons indicated by *, and within marsh seasonal contrast groupings indicated under x-axis (ANOVA contrasts *P* < 0.05).

A total of 23,102 individuals from 27 species or taxa were collected from both natural and restored marsh habitats. (S1 Table). Mean macrofauna density and biomass in the natural marsh varied seasonally, ranging from 4.4 ± 1.8 *n* m^-2^ and 0.05 ± 0.02 g dry wt. m^-2^ in winter 2015 to 101.6 ± 14.3 *n* m^-2^ and 3.6 ± 1.1 g dry wt. m^-2^ in summer 2015 (Fig 5A and 5B) (ANOVA contrasts, *P* ≤ 0.032 for density; *P* < 0.001 for biomass). Mean species richness in the natural marsh also varied among seasons, ranging from 1.5 ± 0.4 in winter 2015 to 5.8 ± 0.6 in summer 2015 (Fig 5C) (ANOVA contrasts, *P* ≤ 0.046). Mean macrofauna abundance in the restored marsh was stable among seasonal sampling periods, ranging from 23.8 ± 7.9 *n* m^-2^ in winter 2015 to 44.6 ± 8.8 *n* m^-2^ in summer 2014 (Fig 5A). However, mean macrofauna biomass and species richness in the restored marsh varied seasonally—with biomass ranging from 0.3 ± 0.1 g dry wt. m^-2^ in winter 2015 to 2.0 ± 0.5 g dry wt. m^-2^ in summer 2014, and richness from 2.8 ± 0.6 in winter 2015 to 4.8 ± 0.4 in summer 2014 (ANOVA contrasts, *P* ≤ 0.003 for biomass; *P* = 0.022 for species richness) (Fig 5B and 5C). Mean macrofauna density, biomass and taxa richness were greater in the restored marsh during summer 2014 (ANOVA contrasts, *P* < 0.001 for density; *P* < 0.001 for biomass; *P* = 0.005 for richness) and winter 2015 (ANOVA contrasts, *P* < 0.001 for density; *P* = 0.031 for biomass; *P* = 0.040 for richness), and greater in the natural marsh during summer 2015 (ANOVA contrasts, *P* = 0.013 for density; *P* < 0.001 for biomass; *P* < 0.001 for richness) (Fig 5A–5C).

**Fig 6:**
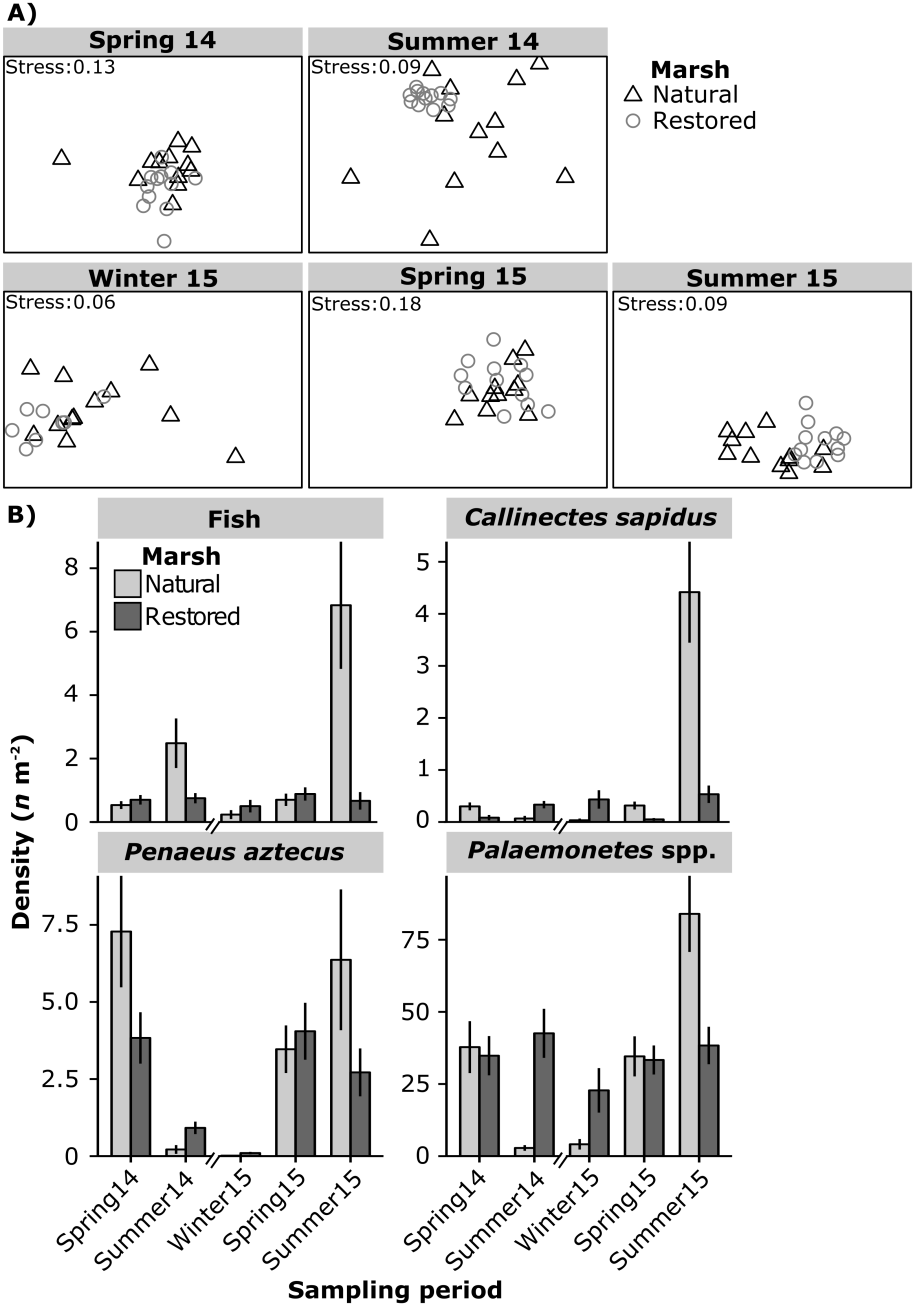
Multivariate community structure. Non-metric multidimensional scaling plots of log(y+1) transformed multivariate macrofauna density data from the natural and restored marshes (A) and mean ± standard error of major taxa density within sampling periods in Nueces Bay, Texas (B).

The results of PERMANOVA showed natural and restored marsh macrofauna community composition were similar during both spring sampling periods and dissimilar during winter 2015 and summer sampling periods (Table 2 and Fig 6A). Homogeneity of group dispersions (betadisper) tests indicated that macrofauna community variability was greater in the natural marsh during summer 2014 and summer 2015 (Table 2 and Fig 6A). *Palaemonetes* spp. was the most numerically abundant consumer in both marsh types; mean densities ranged from 2.8 to 84.0 *n* m^-2^ in the natural marsh and from 27.3 to 42.6 *n* m^-2^ in the restored marsh (Fig 6B). *P*. *aztecus* was the second most abundant consumer species (natural: < 0.1 to 7.3 *n* m^-2^; restored: 0.1 to 4 *n* m^-2^) followed by *C*. *sapidus* (natural: < 0.1 to 4.4 *n* m^-2^; restored: < 0.1 to 0.5 *n* m^-2^) (Fig 6B). Fish densities ranged from 0.2 to 6.8 *n* m^-2^ in the natural marsh and from 0.5 to 0.9 *n* m^-2^ in the restored marsh. Together, *Palaemonetes* spp., *P*. *aztecus* and *C*. *sapidus* composed 95.2% of the community biomass in the natural marsh and 94.7% of the biomass in the restored marsh. The remaining biomass was dominated by fish (S1 Table).

**Table 2:** Multivariate analysis results.

Sampling period	PERMANOVA		Betadisper	
	*t*	*P*_(perm)_	*t*	*P*
Spring 2014	1.34_(1,22)_	0.118	0.84_(1,22)_	0.408
Summer 2014	2.65_(1,22)_	<0.001*	5.59_(1,22)_	<0.001*
Winter 2015	1.98_(1,18)_	0.009*	1.52_(1,18)_	0.146
Spring 2015	0.47_(1,22)_	0.096	0.66_(1,22)_	0.519
Summer 2015	2.22_(1,22)_	0.001*	2.51_(1,22)_	0.020*

Results of PERMANOVA and analyses of multivariate homogeneity of group dispersions (betadisper) comparisons of log(y+1) transformed multivariate community composition data between natural and restored marshes within each sampling period. Numerator and denominator degrees of freedom are given as subscript of *t*-values, significant *P*-values are indicated with

* (α = 0.05).

### Stable isotope analysis

**Fig 7:**
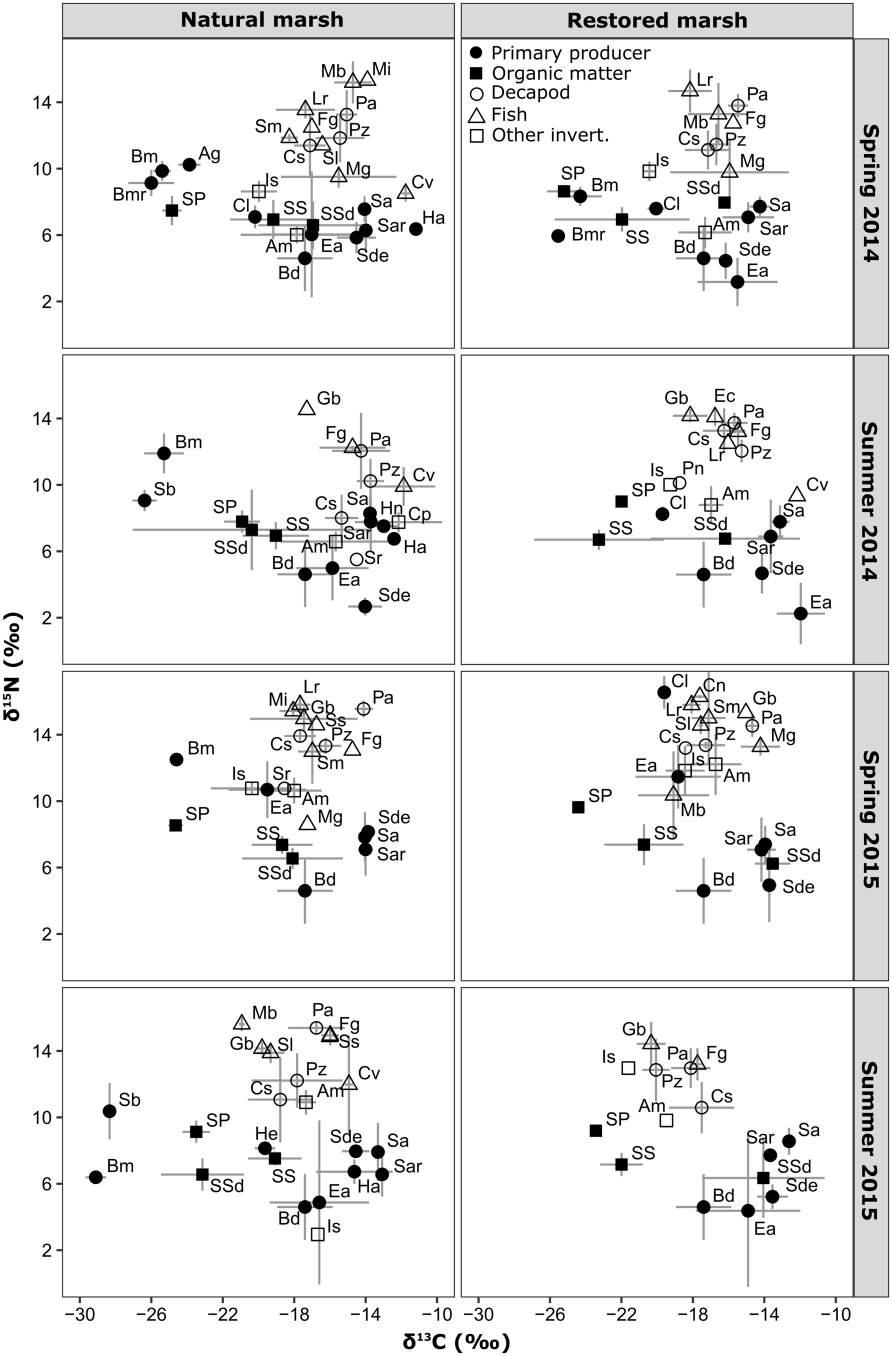
Stable isotope bi-plots. Mean ± standard deviation of δ^13^C and δ^15^N values of potential food resources and consumers in natural and restored marsh sites within sampling periods in Nueces Bay, Texas. Labels representing samples—amphipods: Am, *Avicennia germinans*: Ag, *Batis maritima*: Bm, *Batis maritima* root: Bmr, *Callinectes sapidus*: Cs, *Cerithideopsis pliculosa*: Cp, Filamentous algae: Cl, *Cynoscion nebulosus*: Cn, *Cyprinodon variegatus*: Cv, *Etropus crossotus*: Ec, *Penaeus aztecus*: Pz, *Fundulus grandis*: Fg, *Gobiosoma bosc*: Gb, *H*. *wrightii* epiphyte: He, *Halodule wrightii*: Ha, *Halophila engelmannii*: Hn, Isopod: Is, *Lagodon rhomboides*: Lr. *Menidia beryllina*: Mb, *Micropogonias undulatus*: Mu, *Mugil sp*.: Mg, *Palaemonetes spp*.: Pa, Panopeidae: Pn, *Salicornia bigelovii*: Sb, *Sesarma reticulatum*: Sr, *Spartina alterniflora*: Sa, *Spartina alterniflora* root: Sar, *Spartina* detritus: Sde, *Spartina epiphytic algae*: Ea, SPOM: SP, Sediment detritus: SSd, SSOM: SS, *Strongylura marina*: Sm, *Syngnathus louisianae*: Sl, *Syngnathus scovelli*: Ss. Isotope values for benthic diatoms (Bd) were taken from [25].

No spatial differences in δ^13^C or δ^15^N values were found in primary producers (*Spartina*, *B*. *maritima*, *Spartina* epiphytic microalgae) or in *Spartina* detritus between marsh types over the course of the study (Wilcoxon tests, P > 0.05) (Fig 7 and S2 Table). *Spartina* δ^13^C values varied between sampling periods; ranging from -14.3 ± 0.8‰ in spring 2014 to -13.2 ± 0.5‰ in summer 2015 (summer 2015 > spring 2014/2015; Dunn’s tests, *P* < 0.05). *B*. *maritima* δ^15^N values were lower in summer 2015 (6.3 ± 0.3‰) than in spring 2015 (12.5 ± 0.12‰) or summer 2014 (11.9 ± 1.2‰) (Dunn’s tests, *P* < 0.05). *Spartina* epiphytic microalgae δ^13^C values also varied temporally, ranging from -19.2 ± 2.1‰ in spring 2015 to -13.9 ± 2.6‰ in summer 2014 (summer 2014 > spring 2015; Dunn’s test, *P* = 0.006). The δ^15^N values of *Spartina* epiphytic microalgae were lower in summer 2014 (3.6 ± 2.3‰) than in spring 2015 (11.1 ± 1.7‰) (Dunn’s test, *P* = 0.007).

Over the course of the study, SPOM samples taken in the restored marsh complex had higher δ^15^N values (9.1 ± 0.4‰) than samples taken from natural marsh sites (8.2 ± 0.9‰) (Wilcoxon test, W = 20, *P* = 0.006). SSOM had higher δ^13^C values in the natural marsh (-19.0 ± 1.7‰) than in the restored marsh (-22.0 ± 2.8‰) (Wilcoxon test, W = 216, *P* = 0.001). Sediment macrodetritus in the natural marsh had lower δ^13^C (-19.9 ± 4.6‰) values than in the restored marsh (-15.1 ± 3.1‰) (Wilcoxon test, W = 22, *P* = 0.023). SPOM δ^13^C values varied over the course of the study; with SPOM ranging from -25.0 ± 0.6‰ in spring 2014 to -21.3 ± 1.0‰ in summer 2014 (summer 2014 > spring 2014/2015, summer 2015 > spring 2014; Dunn’s tests, *P* < 0.05).

Mean consumer δ^13^C values ranged from -19.7 to -12.2‰ in the natural marsh and -19.5 to -12.2‰ in the restored marsh. Amphipod δ^13^C and δ^15^N values averaged -17.4 and 8.7‰ in the natural marsh and -17.3 and 9.1‰ in the restored marsh over the course of the study, respectively (Fig 7). Isopods had the lowest average δ^13^C values of all taxa: -19.7‰ in the natural marsh and -19.6‰ in the restored marsh. Mean Isopod δ^15^N values were 8.7‰ in the natural marsh and 11.1‰ in the restored marsh. Mean overall δ^13^C values of major decapods (*Palaemonetes* spp., *P*. *aztecus* and *C*. *sapidus*) ranged from -17.2 to -15.0‰ in the natural marsh and from -17.5 to -16.0‰ in the restored marsh. Their δ^15^N values ranged from 11.1 to 14.0‰ in the natural marsh and from 11.8 to 13.8‰ in the restored marsh (Fig 7). Fish δ^13^C values varied considerably between species. Greatest values were found in *Cyprinodon variegatus* (natural: -13.1‰, restored: -12.2‰) and lowest in *Gobiosoma bosc* (natural: -18.8‰, restored: -18.3‰). Mean fish δ^15^N values also varied widely, with relatively low values in juvenile *Mugil* sp. (10.6‰) and *Cyprinodon variegatus* (10.2‰) in comparison to juvenile *Micropogonias undulatus* (15.4‰) and juvenile *Cynoscion nebulosus* (16.2‰).

Over the course of the study, mean δ^13^C values of *Palaemonetes* spp. were higher in the natural marsh (-15.0 ± 1.5‰) than in the restored marsh (-16.0 ± 1.5‰) (Wilcoxon test, W = 397.5, *P* = 0.010) (Fig 7). *P*. *aztecus* also had greater δ^13^C values in the natural marsh (-15.7 ± 2.0‰) than in the restored marsh (-17.5 ± 1.8‰) (Wilcoxon test, W = 342.5, *P* = 0.006). *C*. *sapidus* δ^13^C values in the natural marsh (-17.2 ± 1.8‰) were similar to those in the restored marsh (-17.1 ± 1.4‰) (Wilcoxon test, W = 57, *P* = 0.872). Among sampling periods, TLs of *Palaemonetes* spp., *P*. *aztecus*, and *C*. *sapidus* were 3.1 ± 0.5, 2.5 ± 0.4 and 2.2 ± 0.7 in the natural marsh, and 3.0 ± 0.3, 2.6 ± 0.3 and 2.5 ± 0.5 in the restored marsh, respectively. There was no overall difference in TL of decapod species between the restored and natural marshes (Wilcoxon tests, *P* < 0.05).

In summer 2014, decapods in the natural marsh had particularly high δ^13^C values and low δ^15^N values in comparison to the decapods in the restored marsh. In the natural marsh, the δ^13^C values of decapods were higher in summer 2014 than during other sampling periods. Mean δ^13^C values of major decapods ranged from -15.4 to -13.7‰ and δ^15^N values ranged from 7.0 to 12.0‰ in the natural marsh during this sampling period (Fig 7). Lowest decapod TLs also occurred during summer 2014 in the natural marsh (*Palaemonetes* spp. = 2.6, *P*. *aztecus* = 2.0, *C*. *sapidus* = 1.4).

### Stable isotope mixing models

**Fig 8:**
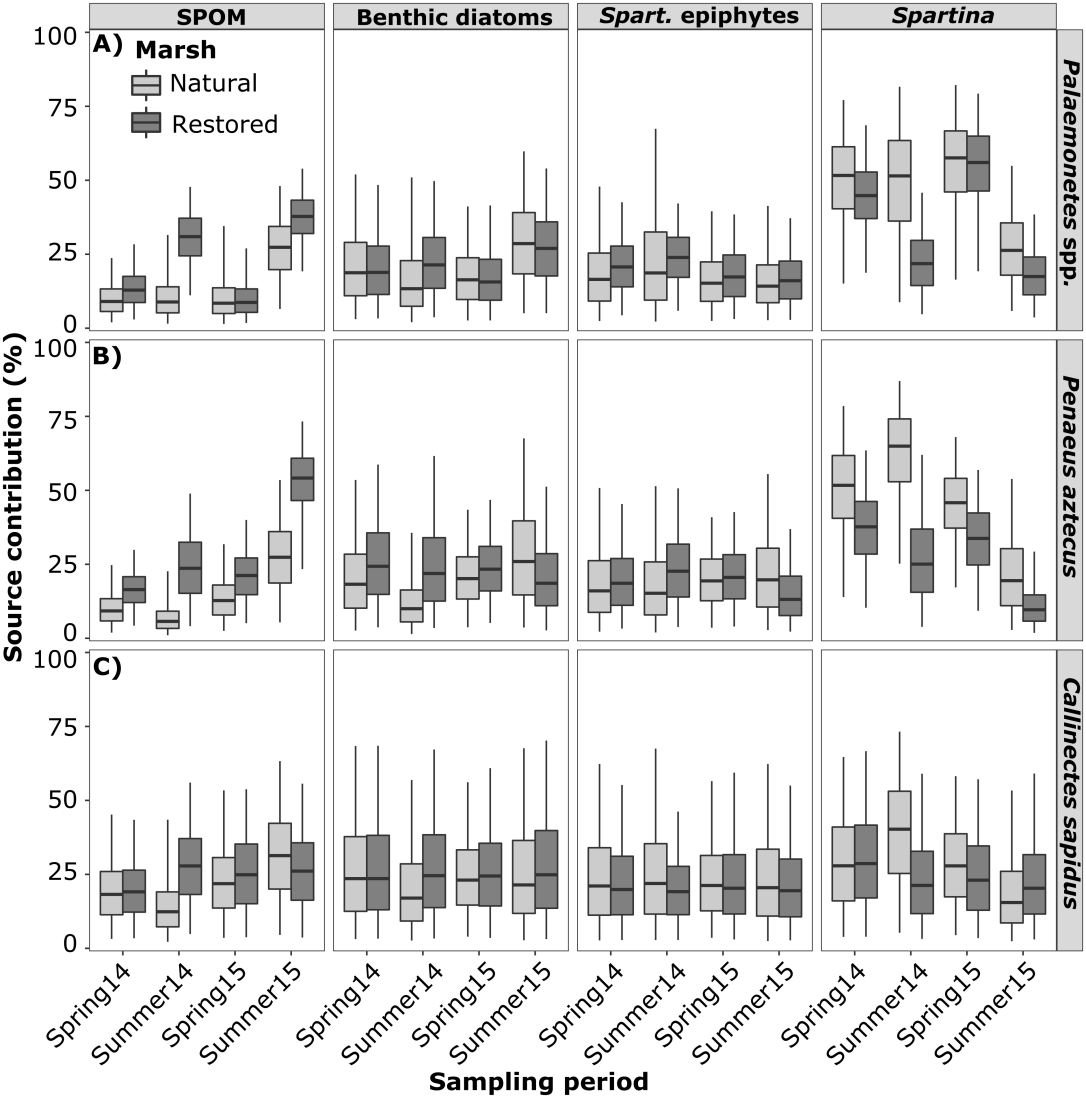
Bayesian stable isotope mixing model results. Estimated proportional contribution of suspended particulate organic matter (SPOM), benthic diatoms, *Spartina* epiphytic microalgae, and *Spartina* to the diets of *Palaemonetes* spp. (A) *P*. *aztecus* (B) and *C*. *sapidus* (C) in spring and summer sampling periods in natural and restored marsh sites. Plots indicate dietary contribution estimate (*posterior* median) with 50% (hinges) and 95% (whiskers) credibility intervals.

SIMM estimated dietary contributions (*posterior* median) to *Palaemonetes* spp. ranged from 26 to 58% for *Spartina* and 8 to 27% for SPOM in the natural marsh, and 17 to 56% for *Spartina* and 9 to 38% for SPOM in the restored marsh (Fig 8A and S3 Table). Contributions to *P*. *aztecus* ranged from 19 to 65% for *Spartina* and 6 to 27% for SPOM in the natural marsh, and 10 to 38% for *Spartina* and 16 to 54% for SPOM in the restored marsh (Fig 8B). Contributions to *C*. *sapidus* ranged from 15 to 40% for *Spartina* and 18 to 31% for SPOM in the natural marsh, and 20 to 29% for *Spartina* and 19 to 29% for SPOM in the restored marsh (Fig 8C). Contributions from benthic diatoms and *Spartina* epiphytic microalgae to decapod diets in the natural marsh ranged from 10 to 29% and 14 to 22%, respectively, and from 16 to 27% and 13 to 24% in the restored marsh, respectively (Fig 8A–8C).

### Hydroperiod influence

**Fig 9:**
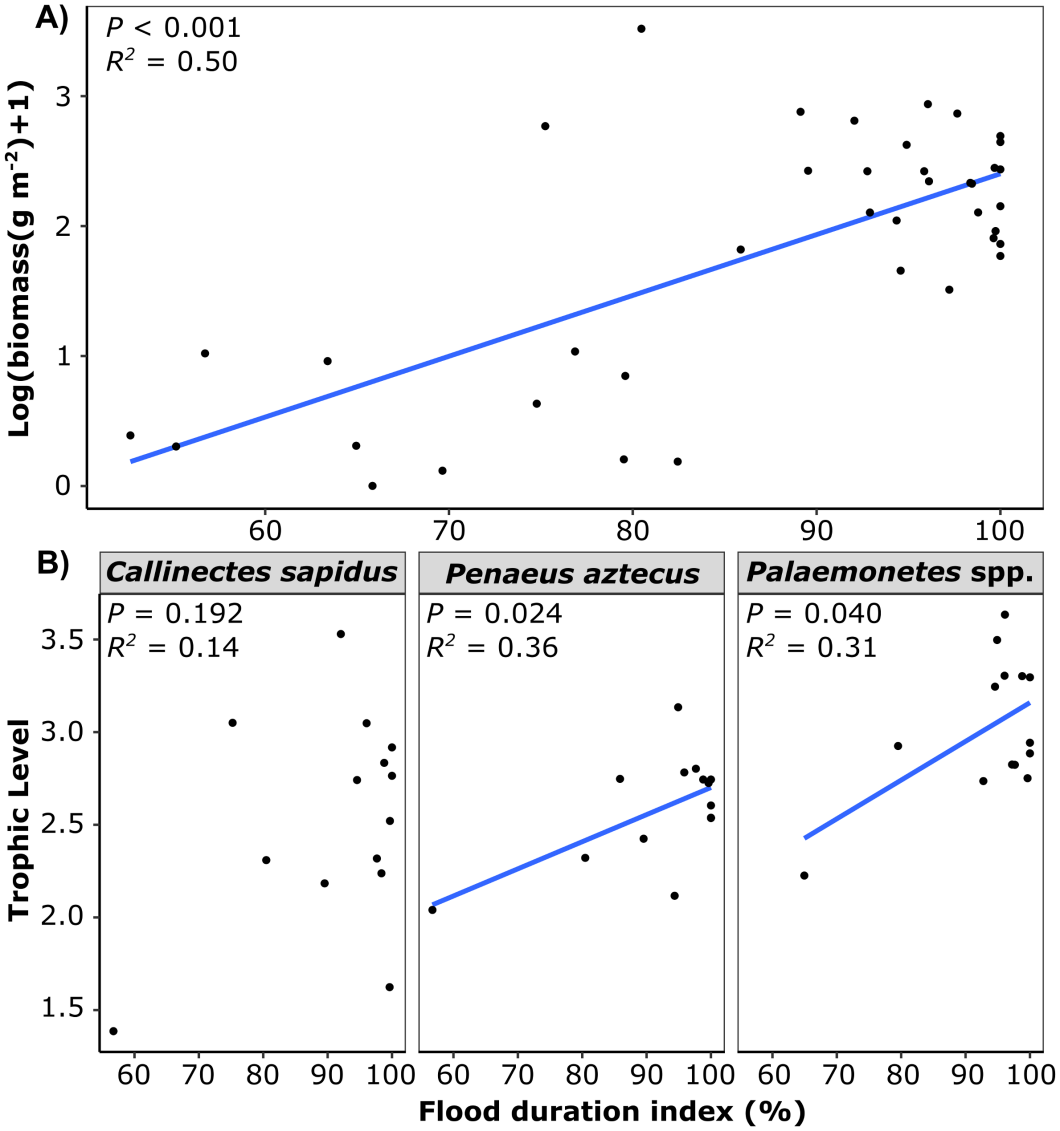
Relationship between hydroperiod, macrofauna biomass, and decapod trophic levels. Relationship between macrofauna dry biomass (g m^-2^) (A), decapod trophic levels (B), and flood duration index in restored and natural sites in spring and summer sampling periods in Nueces Bay, Texas.

Regression models demonstrated a positive relationship between flood duration index and log(y+1) macrofauna biomass in spring and summer sampling periods (F_1,38_ = 37.1, *P* < 0.001, *R*^2^ = 0.50) (Fig 9A). Flood duration index was also positively related to the TLs of *Palaemonetes* spp. (F_1,12_ = 6.8, *P* = 0.040, *R*^2^ = 0.31) and *P*. *aztecus* (F_1,12_ = 10.6, *P* = 0.024, *R*^2^ = 0.36) (Fig 9B). Flooding index did not significantly explain variation in *C*. *sapidus* TLs (F_1,12_ = 2.3, *P* = 0.133, *R*^2^ = 0.18).

## Discussion

### Habitat restoration

*Spartina* density, above- and below-ground biomass were generally similar between the restored and natural marshes, demonstrating recovery within 4 years of marsh construction. However, surface sediment characteristics were substantially different between marsh types. Surface sediment OC, N and macrodetritus content in the restored marsh remained impoverished relative to the natural marsh throughout the study period. These results agree with previous research demonstrating the recovery of restored marsh *Spartina* characteristics (e.g. density, biomass) to natural marsh levels occur relatively quickly (5 to 15 years), while the recovery of restored marsh sediment characteristics occur over much longer timeframes (macro-organic matter: 15 years; OC and N: > 30 years) [41,42].

Although highest macrofauna density, biomass and species richness were observed in the natural marsh during summer 2015, these metrics were similar between marshes or greater in the restored marsh during all other sampling periods. The ability to determine the equivalency of macrofauna community structural characteristics between restored and natural marshes was somewhat complicated by the high degree of temporal variability in the natural marsh used as a reference. Although, macrofauna densities in restored marsh were comparable to those reported in natural marshes in other bays in the Gulf of Mexico: Lavaca Bay (17.65 ± 2.38 *n* m^-2^; [43]) and Galveston Bay (fish = 4.02 ± 0.69 *n* m^-2^, crustaceans = 55.03 ± 8.80 *n* m^-2^; [44]). Between-marsh multivariate similarity followed a comparable trend to that of density, biomass, and species richness. These results support previous research demonstrating the ability of constructed marsh restorations to enhance production of ecologically and economically important macrofauna species in degraded coastal systems [45,4].

### Food web structure

C_3_ and C_4_ marsh macrophytes were easily distinguished based on their δ^13^C values: the relatively low values of *S*. *bigelovii* (-27.4‰) and *B*. *maritima* (-25.8‰) were typical of C_3_ plants, and the relatively high values of *Spartina* (-13.8‰) were typical of C_4_ plants [17]. *S*. *bigelovii* and *Spartina* δ^13^C values were similar to those reported by [25] in the Nueces estuary. SPOM δ^13^C values (-23.6‰) were typical of marine and estuarine phytoplankton [17,46], indicating that the influence of terrestrial organic matter in the water column was relatively low in the study area. The δ^13^C values of sediment macrodetritus in the restored marsh (-15.1‰) indicated that it was largely composed of recently deposited *Spartina* detritus (-14.2‰), whereas the lower δ^13^C values of natural marsh macrodetritus (-19.9‰) were more characteristic of *Spartina* refractory compounds such as lignin, which is generally more ^13^C-depleted than fresh *Spartina* (e.g. -17.0‰ to -17.9‰; [47]), and accumulated in natural marsh sediments over an extended timeframe. A greater influence of ^13^C-depleted mid-marsh halophytes (i.e. C_3_ plants) in natural marsh sediment macrodetritus could have also contributed to its relatively lower δ^13^C values, as these plants were rare in the restored marsh. Isotopic composition of SSOM in the natural marsh (-19.0‰) reflected the δ^13^C values of natural marsh sediment macrodetritus. SSOM δ^13^C values in the restored marsh were lower (-22.0‰), indicating that it was primarily composed of deposited SPOM, and secondarily of benthic microalgae and/or of C_4_ plant detritus.

Mean consumer δ^13^C values in both marshes were within a 7.5‰ range, from -19.7 to -12.2‰, indicating that the secondary production in these habitats was potentially supported by a wide range of resources including *Spartina*, sediment macrodetritus, SPOM, benthic microalgae, and *Spartina* epiphytic microalgae. C_3_ plants were markedly depleted in ^13^C in comparison to consumers, with mean *S*. *bigelovii* and *B*. *maritima* δ^13^C values 6.2‰ and 7.8‰ lower, respectively, than the most ^13^C-depleted consumers (isopods). These results indicate a minimal contribution from C_3_ plants to secondary production in this system, despite the abundance of these plants in natural mid-marsh areas and their potential contribution to natural marsh sediment macrodetritus.

The similar ranges in δ^13^C and δ^15^N values in natural and restored marsh consumers over the course of the study indicate these communities were supported by a similar diversity of basal resources, and had similar food chain lengths. However, *Palaemonetes* spp. and *P*. *aztecus* had slightly greater δ^13^C values in the natural marsh (-15.0 and -15.7‰, respectively) than in the restored marsh (-16.0 and -17.5‰, respectively). This indicates that 1) these consumers significantly rely on SSOM, as SSOM is more enriched in ^13^C in the natural marsh than in the restored marsh, and/or that 2) ^13^C-enriched sources (i.e. *Spartina*, benthic microalgae, *Spartina* epiphytic microalgae, sediment macrodetritus) are used more by these consumers in the natural marsh than in the restored marsh. The lack of systematic difference between marshes in *C*. *sapidus* δ^13^C values may be related to their highly variable and opportunistic feeding strategies [48], leading to greater intra-marsh dietary variability. A potential role of *Spartina* as an important food resource for decapods in both marshes would indeed be consistent with the established paradigm of marsh food webs being largely supported by *Spartina-*derived organic matter [49,50].

Benthic microalgae and *Spartina* epiphytic microalgae are also considered important components of salt marsh food webs [25,51]. Both these algal resources were utilized relatively consistently across inter-seasonal and inter-annual periods. The similar isotopic compositions of *Spartina* epiphytic algae and benthic diatoms during most sampling periods limits the ability of mixing models to discriminate between the use of these two food sources, potentially leading to an underestimated or overestimated contribution of either resource. In combination, models indicated that these microalgal resources were an important food resource for decapods in both marshes. The influence of SPOM generally increased during summer; reflecting an increase in bentho-pelagic coupling, possibly associated with seasonal phytoplankton blooms [52]. SPOM was the most important food source for decapods in both marshes during summer 2015, likely due to an increase in pelagic production associated with greater influx of freshwater (reflected by low salinity in 2015) and associated nutrients earlier in the year [23].

On average, contributions of *Spartina* as a food source to *Palaemonetes* spp. and *P*. *aztecus* were 12% and 19% higher in the natural marsh than in the restored marsh, respectively. In contrast, contributions of SPOM to *Palaemonetes* spp. and *P*. *aztecus* diets were 10% and 15% higher on average in the restored marsh than in the natural marsh, respectively. These differences are likely related to the much greater quantities of macrophyte macrodetritus and/or sediment organic matter accumulated in natural marsh surface sediments in comparison to the recently restored marsh. These results suggest that the relatively impoverished sediments, typical of young constructed marshes, resulted in lower contributions from macrophyte derived organic matter and a greater reliance on pelagic primary production subsidies by important restored marsh community members. Lower flooding durations in natural marsh sites potentially confound the ability to attribute inter-marsh differences in resource use to variations in sediment organic matter versus marsh elevation. However, macrofauna inhabiting marshes with lower flooding frequencies have been shown to consume less *Spartina* derived organic matter than macrofauna in more frequently flooded marshes across a range of systems [21], while the opposite trend was observed in the natural marsh. This supports the conclusion that between-marsh dietary variations were indeed related to differences in sediment organic matter content.

As the quality and abundance of SPOM can vary substantially over time, the reduced availability of macrophyte-derived organic matter in sediments may reduce the food web stability [53] and macrofauna carrying capacity [54] of recently restored marshes in comparison to their natural counterparts. Higher organic matter content in natural marsh sediments may enhance foraging opportunities for macrofauna and infauna prey compared to recently restored marshes [42,55].

The physical design of the restored marsh created marsh edge habitat that was subject to much less exposure and fetch than the natural marsh. Exposure, fetch and associated wind-wave disturbance play an important role in determining the location and condition of marsh edge habitat: *Spartina* in sheltered areas have been shown to occupy lower elevations than in areas exposed to higher wave energy [56,57]. The lower elevations occupied by restored marsh edge *Spartina* indicate the protection provided by the berm and terrace structures may have reduced disturbance associated with wave energy, leading to greater flooding durations.

Flooding duration had a substantial influence in macrofauna communities, explaining 50% of the variation in macrofauna biomass in spring and summer sampling periods. Sampling periods characterized by relatively low flood durations in the natural marsh (i.e. summer 2014, winter 2015) coincided with substantially reduced natural marsh macrofauna density, biomass, and richness. In contrast, the restored marsh macrofauna community was relatively stable, with no significant variation in macrofauna density, biomass or richness between spring and summer sampling periods. These results support the well-documented positive relationship between flood duration and macrofauna use of marsh edge habitat [58,19]. Tidal inundation controls access to important prey that are more abundant (or only available) in marsh edge habitat, such as surface dwelling infauna [59] and *Spartina* epiphyte associated meiofauna [60]. Our results indicated a significant positive relationship between TLs and flood duration index in *Palaemonetes* spp. and *P*. *aztecus*, demonstrating an association between reduced access to marsh edge habitat and lower TLs. These results are consistent with those of Nelson et al. [20], who found a positive relationship between marsh flooding frequency and *Fundulus heteroclitus* TLs.

## Conclusion

The restored marsh demonstrated the ability to support structurally similar macrofauna assemblages as the natural marsh relatively rapidly (4 years after construction), and provided critical habitat to economically important fisheries species. The restoration led to the establishment of a food web fueled by similar resource diversity and supported similar decapod TLs as the natural marsh. However, the food web assessment indicated that dominant consumer species (*Palaemonetes* spp. and *P*. *aztecus*) in the restored marsh relied less on *Spartina* derived resources than in the natural marsh, indicating that the magnitude of the trophic linkage between macrophytes and these consumers was diminished. This was probably due to the much lower quantities of organic matter originating from macrophytes in restored marsh sediments. The accessibility of the marsh to motile consumers, related to marsh edge elevation and hydroperiod dynamics, influenced macrofauna community structure and food resource use, as lower marsh edge flooding durations were associated with reduced macrofauna biomass and dominant consumer TLs. This study highlights the important influence of sediment organic matter and habitat elevation on the functioning of a constructed marsh, with important implications for habitat restoration. This study examined the recovery of a single recently constructed marsh complex and does not account for potential between-system variability. As a result, the ability to make general inferences about the overall effect of marsh restoration on community structure and functions from these results may be limited. Further research on the functioning of constructed marshes across regional scales and at different stages of successional development (i.e. age) is necessary to fully appreciate the ability of constructed marshes to achieve long term functional recovery goals.

## Supporting information

S1 Table Species density and biomass in natural and restored marshes. Total catch (*n*), species density (mean ± standard error, *n* m^-2^) and dry weight biomass (mean ± standard error, g m^-2^) in natural and restored marsh edge habitat in Nueces Bay, Texas.
(DOCX)

S2 Table Stable isotope data from natural and restored marshes. δ^13^C and δ^15^N values (mean ± standard deviation) of potential food sources and consumers from restored and natural marsh sites during spring and summer sampling periods of 2014 and 2015 in Nueces Bay, Texas. If sample size (*n*) differs between elements, sample size for δ^15^N measurement is given after comma.
(DOCX)

S3 Table Bayesian stable isotope mixing model results. Estimated source dietary contribution (%) with upper/lower bounds of 95% credibility intervals (in parentheses) for decapod consumers from natural and restored marshes over the course of the study.
(DOCX)
