# Supplementary material for: Ecological structure and function in a restored versus natural salt marsh
Source: PLoS One. 2017 Dec 19;12(12):e0189871. doi: 10.1371/journal.pone.0189871 (PMC5736197; doi:10.1371/journal.pone.0189871)
Supplement: S1 Table — Total catch (n), species density (mean ± standard error, n m-2) and dry weight biomass (mean ± standard error, g m-2) in natural and restored marsh edge habitat in Nueces Bay, Texas. (DOCX) [file pone.0189871.s001.docx]

|  | **Natural marsh** | | | **Restored marsh** | | |
| --- | --- | --- | --- | --- | --- | --- |
| **Species** | ***n*** | ***n* m^-2^ ±SE** | **g m^-2^ ±SE** | ***n*** | ***n* m^-2^ ±SE** | **g m^-2^ ±SE** |
| Decapods |  |  |  |  |  |  |
| *Palaemonetes spp.* | 9797 | 32.66±5.13 | 0.74±0.11 | 10312 | 34.37±3.15 | 0.84±0.08 |
| *Penaeus aztecus* | 1041 | 3.47±0.7 | 0.52±0.09 | 697 | 2.32±0.35 | 0.32±0.05 |
| *Callinectes sapidus* | 308 | 1.03±0.29 | 0.28±0.18 | 86 | 0.29±0.06 | 0.23±0.12 |
| *Sesarma reticulatum* | 2 | 0.01±<0.01 | <0.01±<0.01 | 0 | 0.00 | 0.00 |
| *Panopeidae* | 0 | 0.00 | 0.00 | 1 | <0.01±<0.01 | <0.01±<0.01 |
| Fish |  |  |  |  |  |  |
| *Lucania parva* | 300 | 1±0.42 | 0.01±<0.01 | 0 | 0.00 | 0.00 |
| *Gobiosoma bosc* | 100 | 0.33±0.1 | 0.01±<0.01 | 96 | 0.32±0.08 | 0.01±<0.01 |
| *Cyprinodon variegatus* | 86 | 0.29±0.12 | 0.01±<0.01 | 5 | 0.02±0.01 | <0.01±<0.01 |
| *Lagodon rhomboides* | 76 | 0.25±0.1 | 0.02±0.01 | 50 | 0.17±0.04 | 0.04±0.02 |
| *Fundulus grandis* | 24 | 0.08±0.03 | 0.01±<0.01 | 18 | 0.06±0.02 | 0.01±<0.01 |
| Unidentified larval fish | 16 | 0.05±0.03 | <0.01±<0.01 | 1 | <0.01±<0.01 | <0.01±<0.01 |
| *Micropogonias undulatus* | 10 | 0.03±0.02 | 0.01±<0.01 | 0 | 0.00 | 0.00 |
| *Bairdiella chrysoura* | 6 | 0.02±0.01 | <0.01±<0.01 | 0 | 0.00 | 0.00 |
| *Menidia beryllina* | 6 | 0.02±0.01 | <0.01±<0.01 | 6 | 0.02±0.01 | <0.01±<0.01 |
| *Syngnathus louisianae* | 5 | 0.02±0.01 | <0.01±<0.01 | 8 | 0.03±0.01 | <0.01±<0.01 |
| *Mugil sp.* | 4 | 0.01±0.01 | <0.01±<0.01 | 9 | 0.03±0.01 | <0.01±<0.01 |
| *Fundulus pulvereus* | 3 | 0.01±0.01 | <0.01±<0.01 | 0 | 0.00 | 0.00 |
| *Strongylura marina* | 3 | 0.01±0.01 | <0.01±<0.01 | 3 | 0.01±0.01 | <0.01±<0.01 |
| *Adinia xenica* | 2 | 0.01±0.01 | <0.01±<0.01 | 3 | 0.01±0.01 | <0.01±<0.01 |
| *Leiostomus xanthurus* | 2 | 0.01±0.01 | <0.01±<0.01 | 0 | 0.00 | 0.00 |
| *Syngnathus scovelli* | 2 | 0.01±<0.01 | <0.01±<0.01 | 0 | 0.00 | 0.00 |
| *Centropristis philadelphica* | 1 | <0.01±<0.01 | <0.01±<0.01 | 0 | 0.00 | 0.00 |
| *Cynoscion nebulosus* | 1 | <0.01±<0.01 | <0.01±<0.01 | 2 | 0.01±<0.01 | <0.01±<0.01 |
| *Alpheus heterochaelis* | 0 | 0.00 | 0.00 | 1 | <0.01±<0.01 | <0.01±<0.01 |
| *Etropus crossotus* | 0 | 0.00 | 0.00 | 2 | 0.01±<0.01 | <0.01±<0.01 |
| *Larimus fasciatus* | 0 | 0.00 | 0.00 | 1 | <0.01±<0.01 | <0.01±<0.01 |
| *Membras martinica* | 0 | 0.00 | 0.00 | 4 | 0.01±0.01 | <0.01±<0.01 |
| *Tozeuma carolinense* | 0 | 0.00 | 0.00 | 2 | 0.01±0.01 | <0.01±<0.01 |
