# Supplementary material for: Ecological structure and function in a restored versus natural salt marsh
Source: PLoS One. 2017 Dec 19;12(12):e0189871. doi: 10.1371/journal.pone.0189871 (PMC5736197; doi:10.1371/journal.pone.0189871)
Supplement: S2 Table — δ13C and δ15N values (mean ± standard deviation) of potential food sources and consumers from restored and natural marsh sites during spring and summer sampling periods of 2014 and 2015 in Nueces Bay, Texas. If sample size (n) differs between elements, sample size for δ15N measurement is given after comma. (DOCX) [file pone.0189871.s002.docx]

| **Species / organic matter** | ***n*** | **δ^13^C±SD (‰)** | **δ^15^N±SD (‰)** |
| --- | --- | --- | --- |
| **Natural marsh** |  |  |  |
| **Spring14** |  |  |  |
| Plant |  |  |  |
| *Avicennia germinans* | 2 | -23.9±0.6 | 10.2±0.1 |
| *Batis maritima* | 3 | -25.4±0.5 | 9.9±0.6 |
| *Batis maritima* root | 3 | -26.0±1.3 | 9.1±0.8 |
| *Cladophora* sp. | 2 | -20.2±0.2 | 7.1±0.7 |
| *Halodule wrightii* | 1 | -11.2 | 6.4 |
| *Spartina alterniflora* | 3 | -14.1±0.3 | 7.6±0.8 |
| *Spartina alterniflora* root | 3 | -14.0±0.4 | 6.3±1.4 |
| *Spartina* detritus | 3 | -14.5±1.1 | 5.8±0.9 |
| *Spartina* epiphytic algae | 5 | -17.0±2.8 | 6.0±3.8 |
| OM pool |  |  |  |
| SPOM | 4 | -24.8±0.5 | 7.5±0.9 |
| SSOM | 4 | -19.2±2.4 | 6.9±1.2 |
| Sediment macrodetritus | 2,4 | -16.9±3.0 | 6.6±1.4 |
| Other invertebrates |  |  |  |
| Amphipod | 3 | -17.9±3.1 | 6.0±0.5 |
| Isopoda | 3 | -20.0±1.0 | 8.6±0.6 |
| Decapod |  |  |  |
| *Callinectes sapidus* | 3 | -17.1±0.9 | 11.4±1.9 |
| *Penaeus aztecus* | 6 | -15.4±1.4 | 11.8±1.4 |
| *Palaemonetes* spp. | 6 | -15.1±0.6 | 13.3±1.5 |
| Fish |  |  |  |
| *Cyprinodon variegatus* | 2 | -11.8±0.2 | 8.5±0.5 |
| *Fundulus grandis* | 1 | -17.0 | 12.5 |
| *Lagodon rhomboides* | 2 | -17.4±1.7 | 13.5±0.5 |
| *Menidia beryllina* | 3 | -14.7±1.0 | 15.2±1.3 |
| *Micropogonias undulatus* | 1 | -13.9 | 15.3 |
| *Mugil* sp. | 3 | -15.5±3.2 | 9.5±0.6 |
| *Strongylura marina* | 2 | -18.3±0.5 | 11.9±0.2 |
| *Syngnathus louisianae* | 1 | -16.4 | 11.4 |
| **Summer14** |  |  |  |
| Plant |  |  |  |
| Batis maritima | 3 | -25.3±1.1 | 11.9±1.2 |
| Halodule wrightii | 3 | -12.4±0.2 | 6.7±0.1 |
| *Halophila engelmannii* | 2 | -13.0±0.9 | 7.5±0.1 |
| *Salicornia bigelovii* | 3 | -26.4±0.7 | 9.0±0.6 |
| *Spartina alterniflora* | 3 | -13.8±0.3 | 8.3±0.9 |
| *Spartina alterniflora* root | 3 | -13.7±0.5 | 7.8±1.8 |
| *Spartina* detritus | 3 | -14.0±0.9 | 2.7±0.5 |
| *Spartina* epiphytic algae | 3 | -15.9±2.0 | 5.0±1.9 |
| OM pool |  |  |  |
| SPOM | 4 | -20.9±1.0 | 7.8±0.7 |
| SSOM | 4 | -19.0±1.9 | 6.9±0.8 |
| Sediment macrodetritus | 4 | -20.4±6.7 | 7.3±2.4 |
| Other invertebrates |  |  |  |
| Amphipod | 2 | -15.7±3.0 | 6.6±0.6 |
| *Cerithideopsis pliculosa* | 2 | -12.2±2.4 | 7.7±0.6 |
| Decapod |  |  |  |
| *Callinectes sapidus* | 3 | -15.4±0.9 | 8.0±1.4 |
| *Penaeus aztecus* | 6 | -13.7±0.8 | 10.2±1.3 |
| *Palaemonetes* spp. | 6 | -14.3±1.6 | 12.0±2.3 |
| *Sesarma reticulatum* | 1 | -14.5 | 5.5 |
| Fish |  |  |  |
| *Cyprinodon variegatus* | 3 | -11.9±1.8 | 9.9±1.2 |
| *Fundulus grandis* | 3 | -14.7±1.9 | 12.2±0.1 |
| *Gobiosoma bosc* | 1 | -17.3 | 14.5 |
| **Spring15** |  |  |  |
| Plant |  |  |  |
| *Batis maritima* | 3 | -24.6±0.1 | 12.5±0.1 |
| *Spartina alterniflora* | 3 | -14.0±<0.1 | 7.8±1.5 |
| *Spartina alterniflora* root | 4 | -14.0±0.4 | 7.1±1.6 |
| *Spartina* detritus | 2 | -13.9±0.2 | 8.1±0.0 |
| *Spartina* epiphytic algae | 3 | -19.5±2.2 | 10.7±1.7 |
| OM pool |  |  |  |
| SPOM | 4 | -24.6±0.4 | 8.5±0.3 |
| SSOM | 4 | -18.7±1.7 | 7.4±0.5 |
| Sediment macrodetritus | 3 | -18.1±2.8 | 6.5±0.6 |
| Other invertebrates |  |  |  |
| Amphipod | 3 | -18.0±1.5 | 10.6±0.8 |
| Isopoda | 3 | -20.4±2.3 | 10.8±0.4 |
| Decapod |  |  |  |
| *Callinectes sapidus* | 3 | -17.7±0.9 | 13.9±0.3 |
| *Penaeus aztecus* | 6 | -16.2±0.9 | 13.3±0.5 |
| *Palaemonetes* spp. | 6 | -14.1±0.5 | 15.6±0.5 |
| *Sesarma reticulatum* | 1 | -18.5 | 10.8 |
| Fish |  |  |  |
| *Fundulus grandis* | 1 | -14.7 | 13.1 |
| *Gobiosoma bosc* | 2 | -17.5±3.0 | 15.0±0.7 |
| *Lagodon rhomboides* | 3 | -17.7±0.6 | 15.8±0.6 |
| *Micropogonias undulatus* | 3 | -18.1±0.8 | 15.4±0.3 |
| *Mugil* sp. | 1 | -17.3 | 8.6 |
| *Strongylura marina* | 2 | -17.0±0.8 | 13.0±1.9 |
| *Syngnathus scovelli* | 1 | -16.8 | 14.6 |
| **Summer15** |  |  |  |
| Plant |  |  |  |
| *Batis maritima* | 3 | -29.1±0.6 | 6.4±0.3 |
| *Halodule* epiphyte | 3 | -19.6±0.6 | 8.1±0.1 |
| *Halodule wrightii* | 3 | -14.6±2.1 | 6.7±0.7 |
| *Salicornia bigelovii* | 3 | -28.3±0.3 | 10.4±1.7 |
| *Spartina alterniflora* | 3 | -13.3±0.3 | 7.9±1.7 |
| *Spartina alterniflora* root | 2 | -13.1±0.1 | 6.6±1.3 |
| *Spartina* detritus | 2 | -14.6±0.8 | 8.0±0.1 |
| *Spartina* epiphytic algae | 3 | -16.6±2.8 | 4.9±4.9 |
| OM pool |  |  |  |
| SPOM | 4 | -23.5±0.8 | 9.1±0.7 |
| SSOM | 4 | -19.1±1.5 | 7.5±0.4 |
| Sediment macrodetritus | 3 | -23.1±2.3 | 6.6±1.0 |
| Other invertebrates |  |  |  |
| Amphipod | 3 | -17.3±0.6 | 10.9±0.7 |
| Isopoda | 1 | -16.7 | 2.9 |
| Decapod |  |  |  |
| *Callinectes sapidus* | 3 | -18.8±1.8 | 11.1±2.6 |
| *Penaeus aztecus* | 5 | -17.8±2.5 | 12.2±1.6 |
| *Palaemonetes* spp. | 5 | -16.8±1.6 | 15.4±0.4 |
| Fish |  |  |  |
| *Cyprinodon variegatus* | 3 | -14.9±0.2 | 12.0±3.0 |
| *Fundulus grandis* | 2 | -16.0±0.5 | 14.9±0.6 |
| *Gobiosoma bosc* | 3 | -19.8±0.3 | 14.1±0.4 |
| *Menidia beryllina* | 2 | -20.9±0.2 | 15.6±0.4 |
| *Syngnathus louisianae* | 2 | -19.3±0.8 | 13.9±0.6 |
| *Syngnathus scovelli* | 1 | -16.0 | 14.9 |
| **Restored marsh** |  |  |  |
| **Spring14** |  |  |  |
| Plant |  |  |  |
| *Batis maritima* | 3 | -24.3±1.2 | 8.3±0.6 |
| *Batis maritima* root | 1,3 | -25.5 | 5.9±0.2 |
| *Cladophora* sp. | 4 | -20.1±0.5 | 7.6±0.3 |
| *Spartina alterniflora* | 3 | -14.2±0.6 | 7.7±0.6 |
| *Spartina alterniflora* root | 3,4 | -14.9±1.4 | 7.1±0.9 |
| *Spartina* detritus | 1,3 | -16.2 | 4.5±1.1 |
| *Spartina* epiphytic algae | 6 | -15.5±2.2 | 3.2±1.4 |
| OM pool |  |  |  |
| SPOM | 2 | -25.2±1.0 | 8.6±0.0 |
| SSOM | 4 | -22.0±3.8 | 6.9±0.7 |
| Sediment macrodetritus | 1 | -16.2 | 8.0 |
| Other invertebrates |  |  |  |
| Amphipod | 3 | -17.3±1.5 | 6.2±0.9 |
| Isopoda | 2 | -20.4±0.3 | 9.8±0.6 |
| Decapod |  |  |  |
| *Callinectes sapidus* | 3 | -17.2±1.3 | 11.1±1.2 |
| *Penaeus aztecus* | 6 | -16.7±0.7 | 11.5±1.3 |
| *Palaemonetes* spp. | 6 | -15.5±0.6 | 13.8±0.7 |
| Fish |  |  |  |
| *Fundulus grandis* | 1 | -15.7 | 12.7 |
| *Lagodon rhomboides* | 3 | -18.2±1.2 | 14.7±1.3 |
| *Menidia beryllina* | 2 | -16.6±1.7 | 13.3±1.9 |
| *Mugil* sp. | 3 | -15.9±3.3 | 9.8±1.9 |
| **Summer14** |  |  |  |
| Plant |  |  |  |
| *Cladophora* sp. | 3 | -19.7±0.1 | 8.2±0.4 |
| *Spartina alterniflora* | 2 | -13.1±0.5 | 7.8±1.0 |
| *Spartina alterniflora* root | 3 | -13.6±0.7 | 6.9±2.2 |
| *Spartina* detritus | 2 | -14.1±0.2 | 4.7±1.2 |
| *Spartina epiphytic* algae | 3 | -12.0±1.3 | 2.2±1.9 |
| OM pool |  |  |  |
| SPOM | 2 | -22.0±0.3 | 9.0±0.1 |
| SSOM | 4 | -23.3±3.6 | 6.7±0.6 |
| Sediment macrodetritus | 4 | -16.2±4.2 | 6.8±0.3 |
| Other invertebrates |  |  |  |
| Amphipod | 3 | -17.0±0.7 | 8.8±1.1 |
| Isopoda | 1 | -19.3 | 10.0 |
| Decapod |  |  |  |
| *Callinectes sapidus* | 3 | -16.3±1.2 | 13.3±1.3 |
| *Penaeus aztecus* | 3 | -15.3±0.0 | 12.0±0.7 |
| *Palaemonetes* spp. | 6 | -15.7±0.8 | 13.7±0.6 |
| Panopeidae | 1 | -18.7 | 10.1 |
| Fish |  |  |  |
| *Cyprinodon variegatus* | 1 | -12.2 | 9.3 |
| *Etropus crossotus* | 2 | -16.8±0.0 | 14.1±0.6 |
| *Fundulus grandis* | 3 | -15.5±0.3 | 13.2±0.9 |
| *Gobiosoma bosc* | 3 | -18.2±1.0 | 14.2±0.4 |
| *Lagodon rhomboides* | 1 | -16.0 | 12.5 |
| **Spring15** |  |  |  |
| Plant |  |  |  |
| *Cladophora* sp. | 3 | -19.6±0.2 | 16.5±1.0 |
| *Spartina alterniflora* | 3 | -14.0±0.1 | 7.4±1.1 |
| *Spartina alterniflora* root | 3 | -14.2±0.8 | 7.1±1.9 |
| *Spartina* detritus | 1 | -13.4 | 3.4 |
| *Spartina* epiphytic algae | 3 | -18.8±2.4 | 11.5±1.9 |
| OM pool |  |  |  |
| SPOM | 2 | -24.4±0.2 | 9.6±0.0 |
| SSOM | 4 | -20.7±2.2 | 7.4±1.2 |
| Sediment macrodetritus | 2 | -13.5±1.0 | 6.2±0.2 |
| Other invertebrates |  |  |  |
| Amphipod | 3 | -16.7±1.4 | 12.2±1.9 |
| Isopoda | 3,2 | -18.4±1.1 | 11.8±1.5 |
| Decapod |  |  |  |
| *Callinectes sapidus* | 1 | -18.4 | 13.2 |
| *Penaeus aztecus* | 6 | -17.3±1.1 | 13.4±0.2 |
| *Palaemonetes* spp. | 6 | -14.7±0.3 | 14.5±0.7 |
| Fish |  |  |  |
| *Cynoscion nebulosus* | 2 | -17.6±0.3 | 16.3±0.1 |
| *Gobiosoma bosc* | 1 | -15.0 | 15.3 |
| *Lagodon rhomboides* | 3 | -18.1±0.5 | 15.8±0.5 |
| *Menidia beryllina* | 3 | -19.1±2.0 | 10.4±2.6 |
| *Mugil* sp. | 3 | -14.2±1.1 | 13.3±0.5 |
| *Strongylura marina* | 2 | -17.1±0.9 | 15.0±2.8 |
| *Syngnathus louisianae* | 3 | -17.6±0.1 | 14.6±0.5 |
| **Summer15** |  |  |  |
| Plant |  |  |  |
| *Spartina alterniflora* | 3 | -12.6±0.3 | 8.6±0.8 |
| *Spartina alterniflora* root | 3 | -13.7±0.2 | 7.7±0.3 |
| *Spartina* detritus | 3 | -13.5±0.9 | 5.2±0.7 |
| *Spartina* epiphytic algae | 3 | -14.9±2.9 | 4.4±4.6 |
| OM pool |  |  |  |
| SPOM | 2 | -23.4±0.0 | 9.2±0.4 |
| SSOM | 4 | -22.0±1.2 | 7.2±0.7 |
| Sediment macrodetritus | 2 | -14.1±3.4 | 6.3±2.4 |
| Other invertebrates |  |  |  |
| Amphipod | 1 | -19.5 | 9.8 |
| Isopoda | 1 | -21.6 | 13.0 |
| Decapod |  |  |  |
| *Callinectes sapidus* | 3 | -17.5±1.8 | 10.6±1.5 |
| *Penaeus aztecus* | 5 | -20.1±0.8 | 12.9±1.9 |
| *Palaemonetes* spp. | 6 | -18.1±1.1 | 13.0±1.2 |
| Fish |  |  |  |
| *Fundulus grandis* | 2 | -17.7±0.3 | 13.2±1.0 |
| *Gobiosoma bosc* | 2 | -20.3±0.8 | 14.4±1.3 |
