# Supplementary material for: Ecological structure and function in a restored versus natural salt marsh
Source: PLoS One. 2017 Dec 19;12(12):e0189871. doi: 10.1371/journal.pone.0189871 (PMC5736197; doi:10.1371/journal.pone.0189871)
Supplement: S3 Table — Estimated source dietary contribution (%) with upper/lower bounds of 95% credibility intervals (in parentheses) for decapod consumers from natural and restored marshes over the course of the study. (DOCX) [file pone.0189871.s003.docx]

| **Sampling period** | SPOM | | Benthic diatoms | | *Spartina* epiphytes | | *Spartina* |
| --- | --- | --- | --- | --- | --- | --- | --- |
| **Natural Marsh** | |  | |  | |  | |
| *Palaemonetes* spp. | |  | |  | |  | |
| spring14 | 9(2/24) | | 19(3/52) | | 16(2/48) | | 52(15/77) |
| summer14 | 9(2/32) | | 13(2/51) | | 19(2/67) | | 51(9/82) |
| spring15 | 8(1/35) | | 16(3/41) | | 15(2/40) | | 58(16/82) |
| summer15 | 27(7/48) | | 29(5/60) | | 14(3/41) | | 26(6/55) |
| *Penaeus aztecus* | |  | |  | |  | |
| spring14 | 9(2/25) | | 18(3/54) | | 16(2/51) | | 52(14/79) |
| summer14 | 6(1/23) | | 10(1/36) | | 15(2/51) | | 65(25/87) |
| spring15 | 13(2/32) | | 20(4/43) | | 19(4/41) | | 46(17/68) |
| summer15 | 27(5/53) | | 26(4/68) | | 20(3/56) | | 19(3/54) |
| *Callinectes sapidus* | |  | |  | |  | |
| spring14 | 18(3/45) | | 24(3/68) | | 21(3/62) | | 28(4/65) |
| summer14 | 12(2/43) | | 17(3/57) | | 22(3/68) | | 40(5/73) |
| spring15 | 22(4/53) | | 23(4/56) | | 21(4/57) | | 28(4/58) |
| summer15 | 31(5/63) | | 21(3/68) | | 20(2/62) | | 15(2/53) |
| **Restored Marsh** | |  | |  | |  | |
| *Palaemonetes* spp. | |  | |  | |  | |
| spring14 | 13(3/28) | | 19(3/48) | | 21(4/43) | | 45(19/69) |
| summer14 | 31(11/48) | | 21(4/50) | | 24(6/42) | | 22(5/46) |
| spring15 | 9(2/27) | | 16(3/41) | | 17(3/38) | | 56(19/79) |
| summer15 | 38(19/54) | | 27(5/54) | | 16(3/37) | | 17(4/38) |
| *Penaeus aztecus* | |  | |  | |  | |
| spring14 | 16(4/30) | | 24(4/59) | | 19(3/45) | | 38(10/64) |
| summer14 | 24(4/49) | | 22(3/62) | | 23(4/51) | | 25(4/62) |
| spring15 | 21(5/40) | | 23(5/47) | | 21(4/43) | | 34(9/57) |
| summer15 | 54(23/73) | | 19(3/51) | | 13(2/37) | | 10(2/29) |
| *Callinectes sapidus* | |  | |  | |  | |
| spring14 | 19(3/43) | | 24(3/68) | | 20(3/55) | | 29(4/67) |
| summer14 | 28(5/56) | | 25(3/67) | | 19(3/46) | | 21(3/59) |
| spring15 | 25(4/54) | | 24(4/61) | | 20(3/59) | | 23(3/57) |
| summer15 | 26(4/56) | | 25(3/70) | | 19(3/55) | | 20(3/59) |
